# Lysosomal protein turnover contributes to the acquisition of TGFβ-1 induced invasive properties of mammary cancer cells

**DOI:** 10.1186/s12943-015-0313-5

**Published:** 2015-02-15

**Authors:** Ursula Kern, Vladimir Wischnewski, Martin L Biniossek, Oliver Schilling, Thomas Reinheckel

**Affiliations:** Institute of Molecular Medicine and Cell Research, Albert-Ludwigs-University Freiburg, Stefan-Meier-Str. 17, Freiburg, D-79104 Germany; Spemann Graduate School of Biology and Medicine, Albert-Ludwigs-University Freiburg, Freiburg, Germany; Faculty of Biology, Albert-Ludwigs-University Freiburg, Freiburg, Germany; BIOSS Centre for Biological Signalling Studies, Freiburg, Germany; German Cancer Consortium (DKTK), Freiburg, Germany

**Keywords:** Cysteine cathepsins, Epithelial-to-mesenchymal transition, Lysosome, Proteome, Transforming growth factor beta

## Abstract

**Background:**

Normal epithelial cells and carcinoma cells can acquire invasiveness by epithelial-to-mesenchymal transition (EMT), a process of considerable cellular remodeling. The endosomal/lysosomal compartment is a principal site of intracellular protein degradation. Lysosomal cathepsin proteases are secreted during cancer progression. The established pro-metastatic role of specific cysteine cathepsins has until now been ascribed to their contribution to extracellular matrix remodeling. We hypothesized that cysteine cathepsins affect transforming growth factor β-1 (TGFβ-1)-induced EMT of normal and malignant mammary epithelial cells.

**Methods:**

The role of lysosomal proteolysis in TGFβ-1-induced EMT and invasion was investigated in a normal and a novel malignant murine mammary epithelial cell line. The contribution of cysteine cathepsins was determined by addition of the general cysteine cathepsin inhibitor E64d. Hallmarks of EMT were analyzed by molecular- and cell-biologic analyses including real-time cell migration/invasion assays. A quantitative proteome comparison using stable isotopic labeling with amino acids in culture (SILAC) showed the effect of E64d on TGFβ-1 induced proteome changes. Lysosomal patterning and junctional adhesion molecule A (Jam-a) localization and abundance were analyzed by immunofluorescence.

**Results:**

We found increased lysosome activity during EMT of malignant mammary epithelial cells. Cysteine cathepsin inhibition had no effect on the induction of the TGFβ-1-induced EMT program on transcriptional level. Protease inhibition did not affect invasion of TGFβ-1 treated normal mammary epithelial cells, but reduced the invasion of murine breast cancer cells. Remarkably, reduced invasion was also evident if E64d was removed 24 h before the invasion assay in order to allow for recovery of cathepsin activity. Proteome analyses revealed a high abundance of lysosomal enzymes and lysosome-associated proteins in cancer cells treated with TGFβ-1 and E64d. An accumulation of those proteins and of lysosomal vesicles was further confirmed by independent methods. Interestingly, E64d caused lysosomal accumulation of Jam-a, a tight junction component facilitating epithelial cell-cell adhesion.

**Conclusion:**

Our results demonstrate an important role of lysosomal proteolysis in cellular remodeling during EMT and a pivotal contribution of lysosomal cysteine cathepsins to TGFβ-1 induced acquisition of breast cancer cell invasiveness. These findings provide an additional rationale to use cathepsin inhibitors to stall tumor metastasis.

**Electronic supplementary material:**

The online version of this article (doi:10.1186/s12943-015-0313-5) contains supplementary material, which is available to authorized users.

## Introduction

Metastatic spread of breast cancers is responsible for most breast cancer deaths. The first critical step of cancer cells leaving a solid tumor is the loss of epithelial integrity and the gain of migratory and invasive capabilities. Cancer cells can acquire this de-differentiated state through epithelial-to-mesenchymal transition (EMT). EMT as it can be found at the invasive fronts of tumors is referred to as “type-3” EMT in contrast to developmental “type-1” or fibrotic “type-2” EMT [1]. Transforming growth factor beta-1 (TGFβ-1) is a strong inducer of type-3 EMT in mammary cancers [2]. TGFβ-1 induced morphological and functional changes of cells are the result of substantial gene regulation and protein alterations leading to: loss of epithelial cell-cell adhesion and apical-basolateral polarity, change of differentiation markers, acquisition of fibroblastoid shape, reversion of intermediate filaments, gain of cell motility and increased extracellular proteolysis [3]. The complex canonical and non-canonical intracellular TGFβ-1 signal transduction is modified by ligand-induced endocytosis of monoubiquitinylated TGFβ-receptor/ligand complexes [4]. At this point TGFβ-1 signaling meets the endolysosomal compartment (hereafter referred to as lysosomes), which represents the site for processing and degradation of proteins delivered by endocytic and autophagic pathways [5,6]. Cysteine cathepsins constitute the largest group of lysosomal proteases with 11 members in humans, namely: Cathepsin B, C, H, F, K, L, O, S, V, W, and X/Z. Besides their concerted and relatively unspecific hydrolysis of lysosomal cargo, specific target proteins and non-lysosomal functions of these proteases in normal as well as pathologic conditions have been identified [7,8]. There are substantial clinical and cell biological data linking cysteine cathepsins, foremost cathepsin B (Ctsb) and cathepsin L (Ctsl), to cancer progression and metastasis [9]. This concept has recently been strongly supported by crossing and analyzing cathepsin-deficient or -overexpressing mice to transgenic mouse models of human cancers including the MMTV-PyMT model for metastasizing breast cancer [10-12]. Pharmacological cysteine cathepsin inhibition in MMTV-PyMT animal studies showed beneficial therapeutic effects especially in combination therapies [13,14]. Cathepsins can be secreted and their tumorigenic and pro-metastatic functions have been mainly ascribed to their ability to directly degrade extracellular matrix (ECM) proteins or activate an extracellular proteolytic cascade [15,16]. In contrast their association with lysosome-mediated cell death pathways implies an anti-tumorigenic role [17]. Inhibition of cathepsins has also pronounced effects on various intracellular signal transduction cascades [18], yet it is not solved how this is linked to the typical localization of cathepsins in lysosomes. One possibility is a role of cathepsins in balancing of growth factor recycling and degradation, as shown for epidermal growth factor [19], thereby affecting mitogenic phosphorylation cascades [20].

Here we addressed the role of lysosomes and especially lysosomal proteases in TGFβ-1 induced transformation of normal and malignant mammary epithelial cells. Therefore we inhibited cathepsins in TGFβ-1 treated normal murine mammary gland epithelial cells (NMuMG) and cells derived from late stage tumors of the murine MMTV-PyMT breast cancer model. By applying quantitative proteomics and cell biological approaches we show that lysosomal protease inhibition results in accumulation of lysosomes and lysosome-related proteins thereby significantly reducing invasion of TGFβ-1 treated breast cancer cells but not of normal mammary epithelial cells.

## Results

### EMT plasticity and cathepsin expression in normal and malignant mammary epithelial cells in response to TGFβ-1

**Figure 1:**
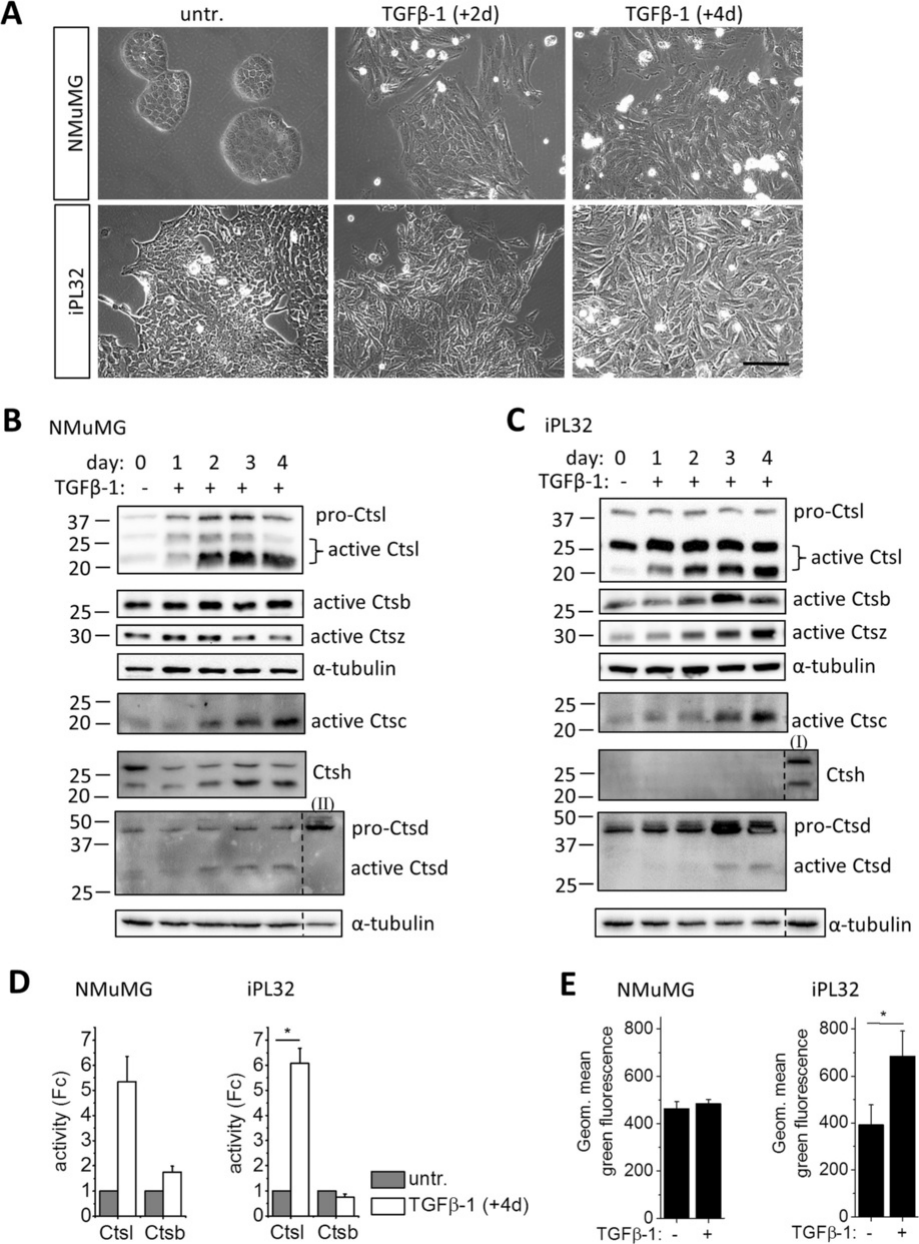
**Increase of lysosomes and lysosomal protease activity during TGFβ-1 induced EMT in NMuMG cells and the MMTV-PyMT breast cancer cell line iPL32.** **(A)** Representative phase contrast images show morphological changes after two and four days TGFβ-1 (2 ng/ml) treatment in NMuMG and iPL32 cells. **(B,C)** Western blot analysis of selected cathepsins in NMuMG **(B)** and iPL32 **(C)** whole cell lysates is shown with α-tubulin as loading control. NMuMG day 0 **(** **I** **)** and iPL32 day 0 **(** **II** **)** were used as positive controls. **(D)** Ctsl and Ctsb activity were measured by z-PheArg-AMC hydrolysis in presence or absence of Ca074 after four days of TGFβ-1 treatment in NMuMG and iPL32 cells, normalized to untreated (untr.) and are shown as the mean ± SEM (n = 3, *p ≤ 0.05 by one sample two tailed t test). **(E)** Abundance of acidic organelles of untreated and four days TGFβ-1 treated NMuMG and iPL32 cells were analyzed by quantitative LysoTracker^TM^ flow cytometry. The mean ± SEM is shown (n = 3, *p ≤ 0.05 by two tailed t-test on independent groups).

Cysteine cathepsin functions during TGFβ-1 induced EMT were analyzed using NMuMG (normal murine mammary gland) cells [21] and a novel MMTV-PyMT breast cancer cell line “iPL32” (immortalized Polyoma Luciferase transgenic), respectively. iPL32 cells were established from primary tumors of a 14 week old MMTV-PyMT-Luc^tg^ mouse. Primary cells which survived the initial crisis were passaged using selective trypsinization to enrich epithelial cells, resulting in the cell line iPL32 (Figure 1A). For experimental induction of EMT cells were treated with TGFβ-1 (2 ng/ml). As shown previously [21], two and four days TGFβ-1 treatment induced cell death in a high number of NMuMG cells and the acquisition of a spindle-like mesenchymal morphology in surviving cells (Figure 1A). In iPL32 cells TGFβ-1 also induced a successive phenotypic transition from an initial epithelial cobblestone-like to a depolarized triangular or spindle-like mesenchymal morphology.

Next we analyzed protein expression and activity of selected cathepsins and quantified acidic organelles in response to TGFβ-1. The active forms of cysteine cathepsins Ctsl, Ctsc, and Ctsh increased during four days of TGFβ-1 treatment in NMuMG cells. The aspartic lysosomal protease Ctsd was hardly detectable (Figure 1B). In iPL32 cells Ctsd, as well as Ctsl, Ctsz and Ctsc active forms increased in response to TGFβ-1, while Ctsh was not detectable (Figure 1C). TGFβ-1 triggered Ctsl but not Ctsb activity in both cell lines (Figure 1D). Quantification of acidic organelles, i.e. the endolysosomal cell compartment, by LysoTracker^TM^ flow cytometry revealed a significant increase of endolysosome staining in iPL32 cells, but not in NMuMG cells (Figure 1E). In summary, increased cathepsin activity in both cell lines pointed towards a role of lysosomal proteolysis during TGFβ-1 induced EMT. The significant increase of acidic organelle staining in iPL32 cells, but not in NMuMG cells, raised the question if lysosomal proteolysis during EMT is more relevant for malignant cells than for normal cells. To explore this idea, human non-small cell lung carcinoma A549 cells were used as an independent model. TGFβ-1 is able to induce EMT in A459 cells (Additional file 1: Figure S1A). Comparable to iPL32 cells TGFβ-1 treatment led to a significant increase of LysoTracker™ signal in A549 cells (Additional file 1: Figure S1B).

### EMT regulation, cell viability and proliferation upon cysteine cathepsin inhibition

Cell morphology, expression of known EMT markers, cell viability, and proliferation upon TGFβ-1 treatment were analyzed in absence or presence of the cell permeable pan-cysteine cathepsin inhibitor E64d in order to determine the role of concerted cysteine cathepsin activity during EMT. NMuMG and iPL32 cells treated with TGFβ-1 + E64d underwent the same morphological changes than cells treated with TGFβ-1 alone (Additional file 2: Figure S2A). Quantitative RT-PCR analysis showed robust and early induction of the key EMT transcription factors snail1 and zeb-1 as well as downregulation of the cell adhesion protein E-cadherin in NMuMG cells (Additional file 2: Figure S2B). In iPL32 cells four days of TGFβ-1 treatment led to a three-fold increase of snail1, but transcription of zeb1 and E-cadherin were not altered (Additional file 2: Figure S2C). In both cell lines E64d did not affect snail1, zeb1 or E-cadherin transcription. In iPL32 cells E-cadherin protein levels were not reduced after four days TGFβ-1 treatment, but at the same time N-cadherin expression was increased (Additional file 2: Figure S2D). Neither E-cadherin nor N-cadherin expression were altered with E64d. In conclusion, TGFβ-1 induced a partial EMT in iPL32 cells in contrast to the induction of a classical EMT in NMuMG cells. Cysteine cathepsin inhibition did not affect the induction of EMT in normal or malignant mouse mammary epithelial cells.

Lactate dehydrogenase (LDH) release was measured as an indicator of cell viability. In addition numbers of living cells were determined. In untreated NMuMG cells release of 5% of total cellular LDH, with a highly significant increase to 15% upon TGFβ-1 treatment was measured (Additional file 2: Figure S2E). In comparison, iPL32 cells showed a significant but smaller increase of LDH release of 11% in untreated to 17% in TGFβ-1 treated cells. In both cell lines LDH release was not changed by E64d treatment. Determination of living cell numbers over six days revealed an anti-proliferative effect of TGFβ-1 on both cell lines, while E64d treatment had no impact on cell numbers (Additional file 2: Figure S2F). Taken together, E64d did not change TGFβ-1 induced proliferation arrest and cell death in both normal as well as malignant mammary epithelial cells.

### Cysteine cathepsin activity contibutes to the acquisition of invasiveness in two TGFβ-1 responsive cancer cell lines

**Figure 2:**
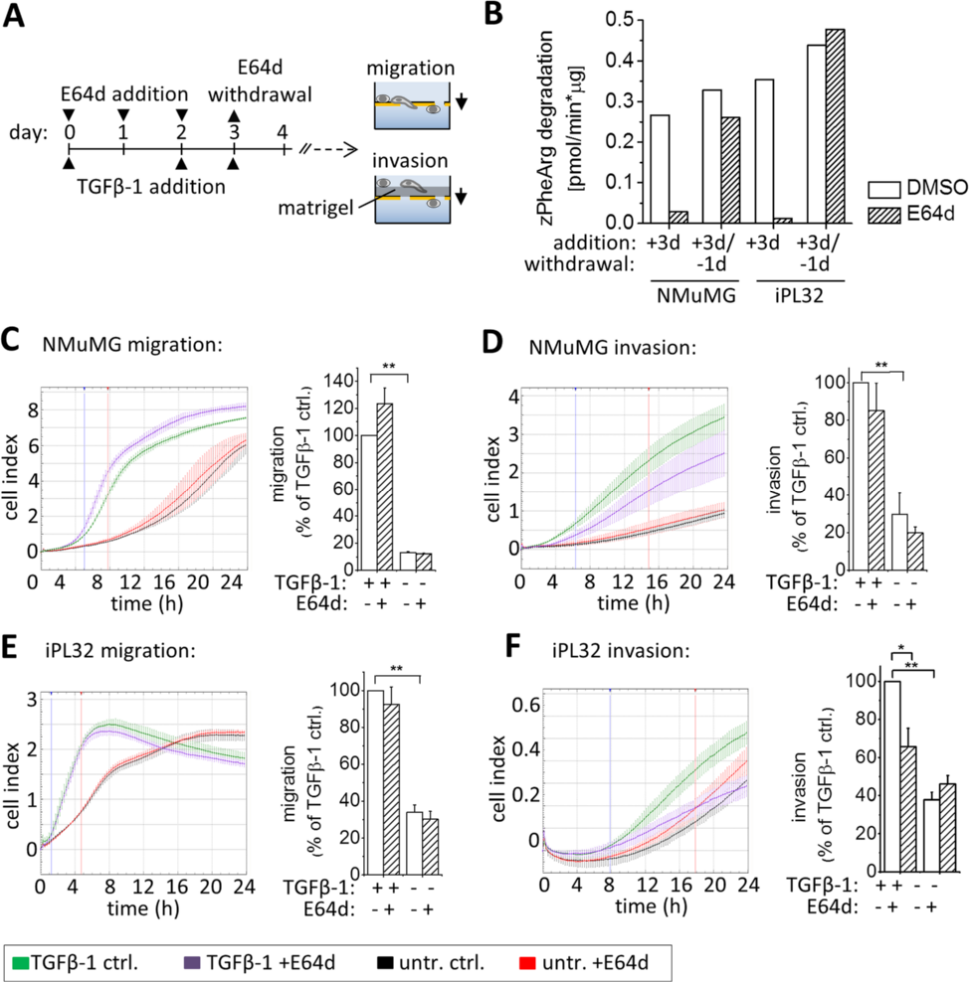
**Inhibition of cysteine cathepsins during EMT impaired TGFβ-1 induced invasiveness of malignant cells. (A)** Experimental setup: Cells were either untreated or treated with TGFβ-1 for four days in presence of E64d or solvent control (“ctrl”) for three days. At day three E64d was removed. At day 4 cells were trypsinized and directed migration or invasion through Cultrex®-coated membranes were analyzed for 24 h by RTCA real time trans-well assays in absence of E64d. **(B)** Cysteine cathepsin activity measured by z-Phe-Arg-AMC hydrolysis in NMuMG and iPL32 whole cell lysates after three days E64d treatment and one day E64d withdrawal. **(C-F)** Migration and Invasion of NMuMG **(C,D)** and iPL32 cells **(E,F)**. Cell indexes as function of time for the triplicates of representative experiments (left panels); statistical analysis of independent experiments (right panels) calculated as the slope of cell index between the time points marked in the time-curves, normalized to “TGFβ-1 ctrl.”, shown as the mean ± SEM (n = 3 for NMuMG and n = 5 for iPL32 cells, *p ≤ 0.05, **p ≤ 0.01).

To investigate a potential role of cysteine cathepsins in the transformation of cells towards a migratory/invasive phenotype, NMuMG and iPL32 cells were treated with TGFβ-1 + E64d for three days for complete cysteine cathepsin inhibition during EMT. E64d was removed at day three to allow re-expression of active cysteine cathepsins within the next 24 h (Figure 2A and B). At day four directed cell migration and invasion were analyzed by xCelligence [22] in absence of TGFβ-1 and E64d for the following 24 h. Untreated NMuMG cells on average showed only 20% of the migration and invasion of TGFβ-1 treated NMuMG cells (Figure 2C and D). E64d had no impact on migration and invasion of NMuMG cells. TGFβ-1 also highly increased migration and invasion of iPL32 cells (Figure 2E and F). On average untreated iPL32 cells showed only 40% of the migratory and invasive capacity of TGFβ-1 treated iPL32 cells. Addition of E64d did not change migration of iPL32 cells (Figure 2E) or invasion of untreated iPL32 cells, but E64d caused a significant 30-40% reduction of invasion of TGFβ-1 treated iPL32 cells (Figure 2F). Also in A549 non-small cell lung carcinoma cells TGFβ-1 significantly induced invasion. When cysteine cathepsins were inhibited during TGFβ-1 treatment, A549 cells showed a tendency towards lower invasion (Additional file 1: Figure S1C). In summary, cysteine cathepsin inhibition specifically impaired TGFβ-1 induced invasion of malignant epithelial cells through a basal membrane-like Cultrex® matrix.

### Cysteine cathepsin activity directly contributes to invasion of TGFβ-1 transformed murine breast cancer cells

**Figure 3:**
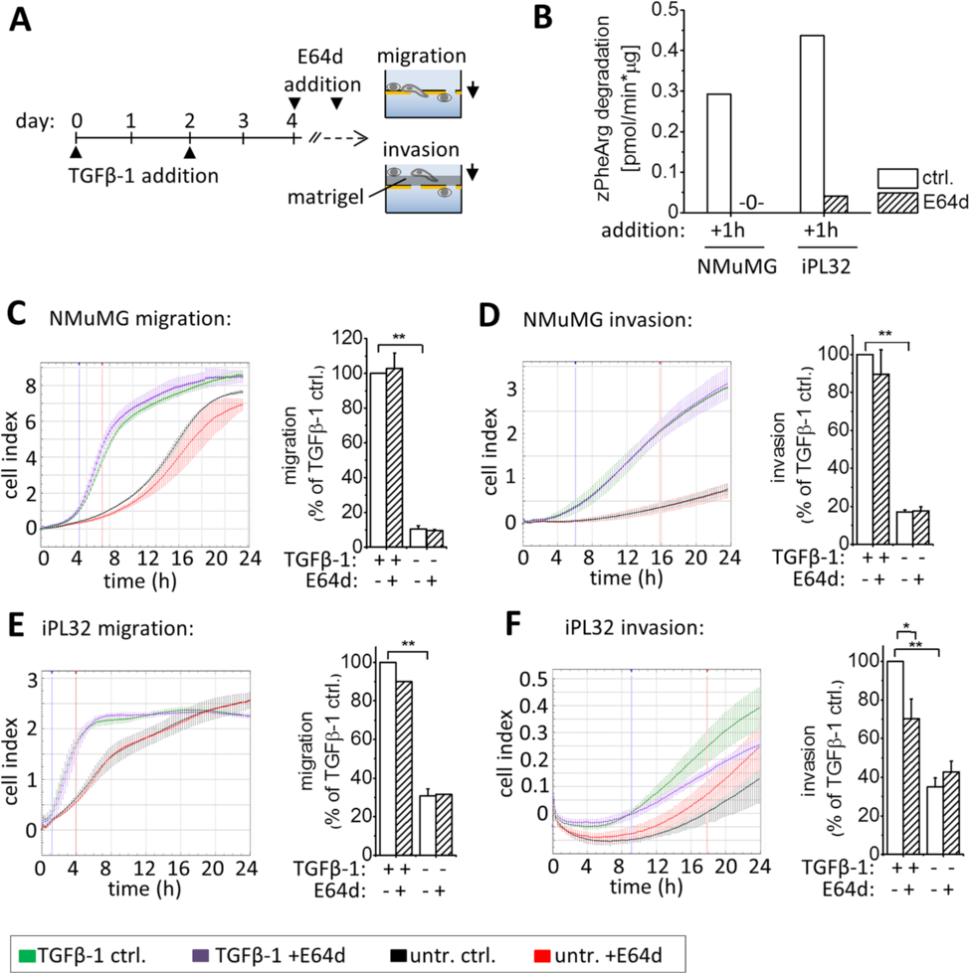
**Inhibition of cysteine cathepsins during migration/invasion reduced invasion of TGFβ-1 transformed malignant cells. (A)** Experimental setup: Cells were treated without or with TGFβ-1 for four days in the absence of E64d. At day four cells were incubated with E64d or solvent control (“ctrl.”) for one hour. Thereafter cells were trypsinized and migration and invasion were analyzed for 24 h by RTCA real time trans-well assays in presence of E64d. **(B)** Cysteine cathepsin activity measured as z-Phe-Arg-AMC hydrolysis in NMuMG and iPL32 whole cell lysates after 1 h E64d treatment. **(C-F)** Migration and invasion of **(C,D)** NMuMG and **(E,F)** iPL32 cells. Graphs show the cell indexes of triplicates of representative experiments (left panels); statistical analysis of independent experiments (right panels) calculated as the slope of cell index between the time points marked in the time-curves, normalized to “+TGFβ-1 ctrl.”, shown as the mean ± SEM (n = 3 for NMuMG and n = 5 for iPL32 cells, *p ≤ 0.05, **p ≤ 0.01).

In numerous studies cysteine cathepsins were shown to directly promote cancer cell invasion by ECM degradation [16]. To confirm the direct contribution of cysteine cathepsins to invasion, cells were treated with TGFβ-1 for four days in absence of E64d. E64d was added 1 h prior to and during the 24 h migration/invasion assays to inhibit cysteine cathepsin activity only during cell migration/invasion (Figure 3A and B). E64d had no direct effect on migration and invasion of NMuMG cells (Figure 3C und D). Migration of iPL32 cells was also not reduced (Figure 3E). Interestingly, only invasion of TGFβ-1 treated but not of untreated iPL32 cells was significantly reduced upon cysteine cathepsin inhibition (Figure 3F). In summary, the results indicate that cysteine cathepsin activity fosters TGFβ-1 induced invasion of malignant cell by promoting type-3 EMT (Figure 2) and in addition by directly promoting invasion of TGFβ-1 transformed cells (Figure 3).

### Effects of cathepsin inhibition on the proteome of TGFβ-1 treated iPL32 breast cancer cells

**Figure 4:**
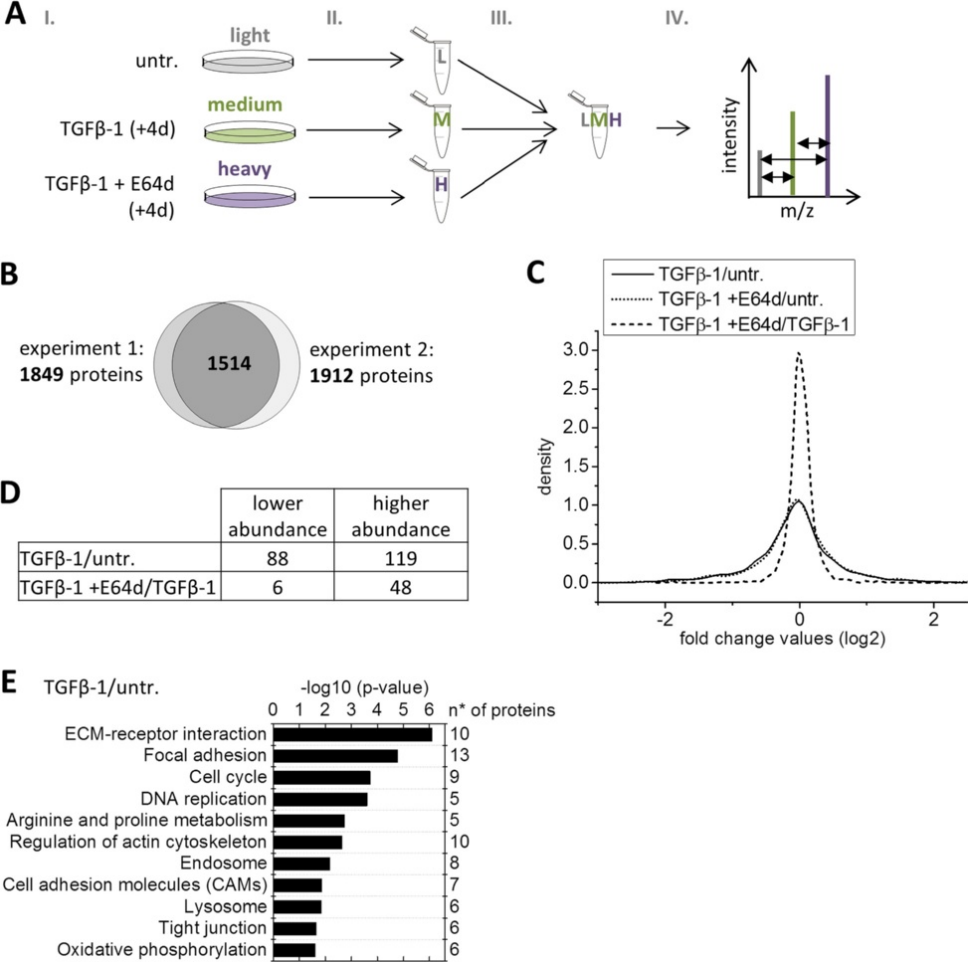
**Quantitative proteome comparison of untreated, TGFβ-1, and TGFβ 1 + E64d treated iPL32 cells. (A)** Workflow: I: Metabolic stable isotopic labeling (SILAC) of untreated cells with light “L”, TGFβ-1 treated cells with medium “M”, and TGFβ-1 + E64d treated cells with heavy “H” amino acids. II: Cell lysis and protein preparation. III: Quantification and combination of samples. IV: Fractionation, protein cleavage by trypsin, and peptide preparation for LC MS/MS analysis. **(B)** Venn diagram showing the total number of proteins identified in each independent experiment and in both experiments. **(C)** Density plot showing the distribution of Fc values (log_2_) for the comparisons of the conditions untreated to TGFβ-1 as well as TGFβ-1 to TGFβ-1 + E64d in experiment 1. **(D)** Number of proteins altered in abundance in both experiments in the proteome comparison of untreated to TGFβ-1 treated cells as well as TGFβ-1 treated to TGFβ-1 + E64d treated cells. log_2_ Fc ≤ 0.58 = less abundant; log_2_ Fc ≥ 0.58 = more abundant. **(E)** KEGG (Kyoto Encyclopedia of Genes and Genomes) pathway enrichment analysis of proteins altered in abundance (log_2_ Fc ≤ or ≥ 0.58) in the quantitative proteome comparison of untreated to TGFβ-1 treated iPL32 cells. Functional pathways significantly enriched (with p ≤ 0.05; ≥ 6 proteins in pathway) in the KEGG analysis are shown.

To gain a comprehensive understanding of TGFβ-1 induced changes during type-3 EMT in iPL32 cells and the contribution of cysteine cathepsins to this process, a quantitative proteome comparison was conducted. Triple stable isotopic labeling with amino acids in cell culture (SILAC) was applied (Figure 4A). Metabolic labeling of untreated (light), TGFβ-1 treated (medium) and TGFβ-1 + E64d treated cells (heavy) was followed by sample preparation and LC MS/MS analysis. Two independent experiments were performed. 1849 proteins were identified in the first, 1912 proteins in the second and 1514 proteins in both experiments (Figure 4B). Alterations of protein abundances were calculated as log_2_ fold change (Fc) values of the TGFβ-1/untreated and TGFβ-1 + E64d/TGFβ-1 ratios. The Fc values of all identified proteins showed a normal distribution (Figure 4C). Proteins with altered abundance of more than 50% in both experiments (log_2_ Fc ≤ −0.58 or ≥ 0.58) were considered to be altered in their protein levels: 88 proteins with lower abundance and 119 proteins with higher abundance upon TGFβ-1 treatment were identified (Figure 4D and Additional file 3: Table S1 and Additional file 4: Table S2). These included known invasion-promoting TGFβ-1 downstream effects, i.e. higher abundance of clusterin, vimentin, tenascin C, and integrin-β1. E64d treatment led to lower abundance of 6 proteins and higher abundance of 48 proteins (Figure 4D and Additional file 5: Table S3). Proteins with altered levels upon TGFβ-1 treatment were clustered using KEGG (Kyoto Encyclopedia of Genes and Genomes) pathway enrichment (Figure 4E). The functional clusters most significantly enriched were known EMT-related processes such as: ECM receptor interaction, focal adhesion, metabolic pathways, cell cycle, DNA replication, regulation of actin cytoskeleton and tight junction. Importantly, the clusters endosome and lysosome were also altered in response to TGFβ-1. The higher abundance of several lysosomal proteins (highlighted in Additional file 4: Table S2) is in line with the increased labeling of TGFβ-1 treated iPL32 cells with the acidotropic LysoTracker™ dye (Figure 1E).

### Accumulation of lysosomes upon cysteine cathepsin inhibition

**Figure 5:**
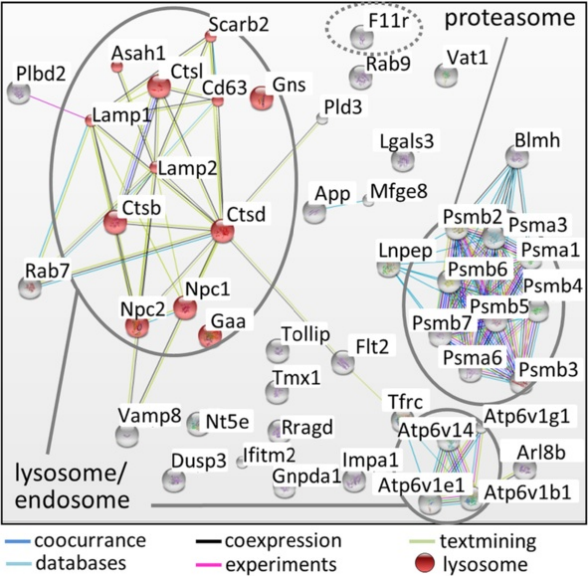
**Proteins more abundant upon cysteine cathepsin inhibition.** The connectivity of all proteins more abundant (log_2_ Fc ≥ 0.58) in the quantitative proteome comparison of TGFβ-1 treated to TGFβ-1 + E64d treated iPL32 cells was determined with STRING (Search Tool for the Retrieval of interacting Genes/Proteins). The three clusters most significantly enriched: lysosome p = 2.3 x 10^−12^, endosome p = 4.6 x 10^−7^, proteasome p = 4.2 x 10^-12^, and Jam-a “f11r” are highlighted. Different line colors indicate the type of evidence for interaction.

**Figure 6:**
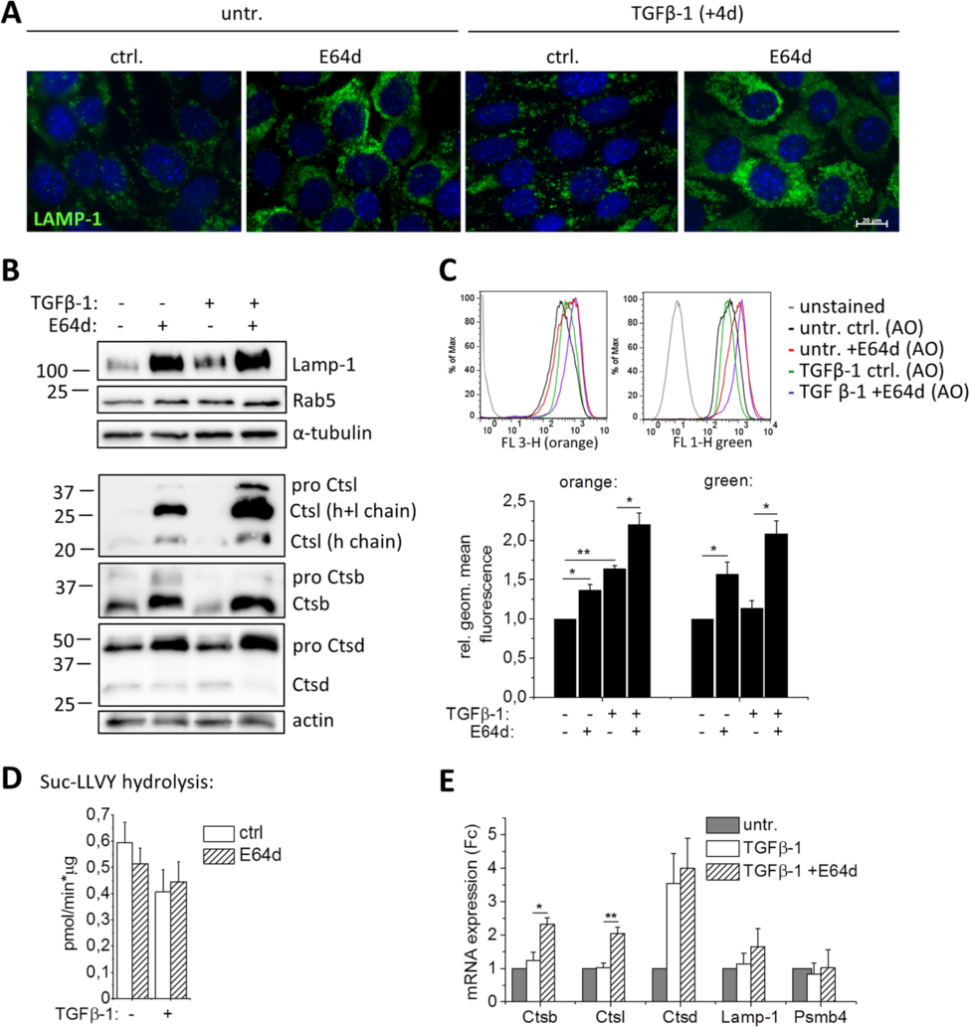
**Accumulation of endosomal/lysosomal proteins and proteasomes upon cysteine cathepsin inhibition. (A)** Representative Lamp-1 immunofluorescence images of untreated −/+E64d and four days TGFβ-1 −/+E64d treated iPL32 cells are shown (Lamp-1: green, Hoechst nuclear stain: blue). **(B)** Lamp-1, Rab5 Ctsl, Ctsb, and Ctsd protein levels were analyzed by Western blot with α-tubulin and actin as loading controls in whole cell lysates of untreated −/+E64d and four days TGFβ-1 −/+E64d treated iPL32 cells. Ctsl heavy (h) and light (l) chains indicate the fully processed mature protease. **(C)** Flow cytometry analysis of Acridine-Orange (AO) stained iPL32 cells pretreated with or without TGFβ-1 −/+E64d for four days: Representative histograms for FL-3 height (orange) and FL-1 height (green) are shown in the upper panel, statistical analysis of independent experiments in the lower panel. Geometric mean orange or green fluorescence was normalized to untreated control cells (n = 3, *p ≤ 0.05, **p ≤ 0.01). **(D)** Proteasome activity of untreated −/+E64d and four days TGFβ-1 −/+E64d treated iPL32 cells was measured by Suc-LLVY-AMC cleavage (n = 3). **(E)** Ctsb, Ctsl, Ctsd, Lamp-1, and proteasome subunit 4 “Psmb4” mRNA levels in untreated and four days TGFβ-1 −/+E64d treated iPL32 cells were analyzed by qRT-PCR. Starting quantity values were normalized to the untreated group. Data are shown as the mean ± SEM (n = 3, *p ≤ 0.05 **p ≤ 0.01 by two sample two sided t-test).

In order to analyze the consequences of cysteine cathepsin inhibition on the proteome changes during type-3 EMT of iPL32 cells, proteins with a higher abundance in TGFβ-1 + E64d compared to TGFβ-1 treated cells were further analyzed. The connectivity of these proteins was determined by the STRING online tool (Figure 5). Noticeably, E64d led to a higher abundance of lysosomal enzymes (i.e. Ctsb, Ctsl, and Ctsd , acid ceramidase, lysosomal alpha glucosidase, and bleomycin hydrolase) and lysosomal membrane proteins (i.e. lysosome associated membrane protein (Lamp) 1–3, Niemann-Pick C1 and C2 protein, and V-type ATPase). To analyze the state of the lysosomal compartment, Lamp-1 immunofluorescence staining was performed (Figure 6A). Four days TGFβ-1 treatment resulted in a higher number of lysosomes and their reorganization from a perinuclear to a diffuse distribution. Independent of TGFβ-1, E64d treatment caused a massive increase of enlarged lysosomes, a phenotype also found in lysosomal storage disorders [23]. Lamp-1 increase in presence of TGFβ-1 and TGFβ-1 independent Lamp-1 amplification with E64d was confirmed by Western blot (Figure 6B). In contrast, expression of the early endosome marker Rab5 was not changed upon E64d treatment, thereby confirming a lysosome specific effect. A higher abundance of Ctsb and Ctsl in cells treated with TGFβ-1 + E64d was confirmed by Western blot and was also found in cells treated with E64d alone (Figure 6B). It should be noted that the “active” cathepsins in this blot are actually inhibited covalently by E64d (which also explains the slight band shifts in the blots). An elevated level of the Ctsd zymogen was detected, while the band of active Ctsd remained unchanged. This indicates that active cysteine cathepsins are needed for Ctsd processing. Lysosomal accumulation was confirmed by Acridine-Orange staining, an acidotropic dye with orange fluorescence at high concentrations and green fluorescence at low concentrations. With TGFβ-1 treatment, an increase in orange fluorescence was detectable confirming an increase of acidic vesicles. E64d led to an increase in orange as well as green fluorescence, which is indicative for a loss of acidic pH in the accumulating lysosomes (Figure 6C). A similar accumulation of lysosomes was found In A549 cells upon E64d treatment (Additional file 1: Figure S1D). Interestingly a higher abundance of proteasome subunits was found in cells treated with E64d. However, proteasome activity measured by cleavage of the fluorogenic peptide Suc-LLVY-AMC was not increased (Figure 6D). The higher abundance of Ctsb and Ctsl could partially be explained by compensational transcriptional upregulation upon their inhibition (Figure 6E). Transcription of Ctsd, Lamp-1 and proteasome subunit 4 (psmb4) was not upregulated, indicating an accumulation of these proteins due to lysosomal protease inhibition. In summary, the results corroborate increased lysosome activity during type-3 EMT and point towards lysosomal storage of undigested material upon cysteine cathepsin inhibition.

### Jam-a expression and lysosomal accumulation

**Figure 7:**
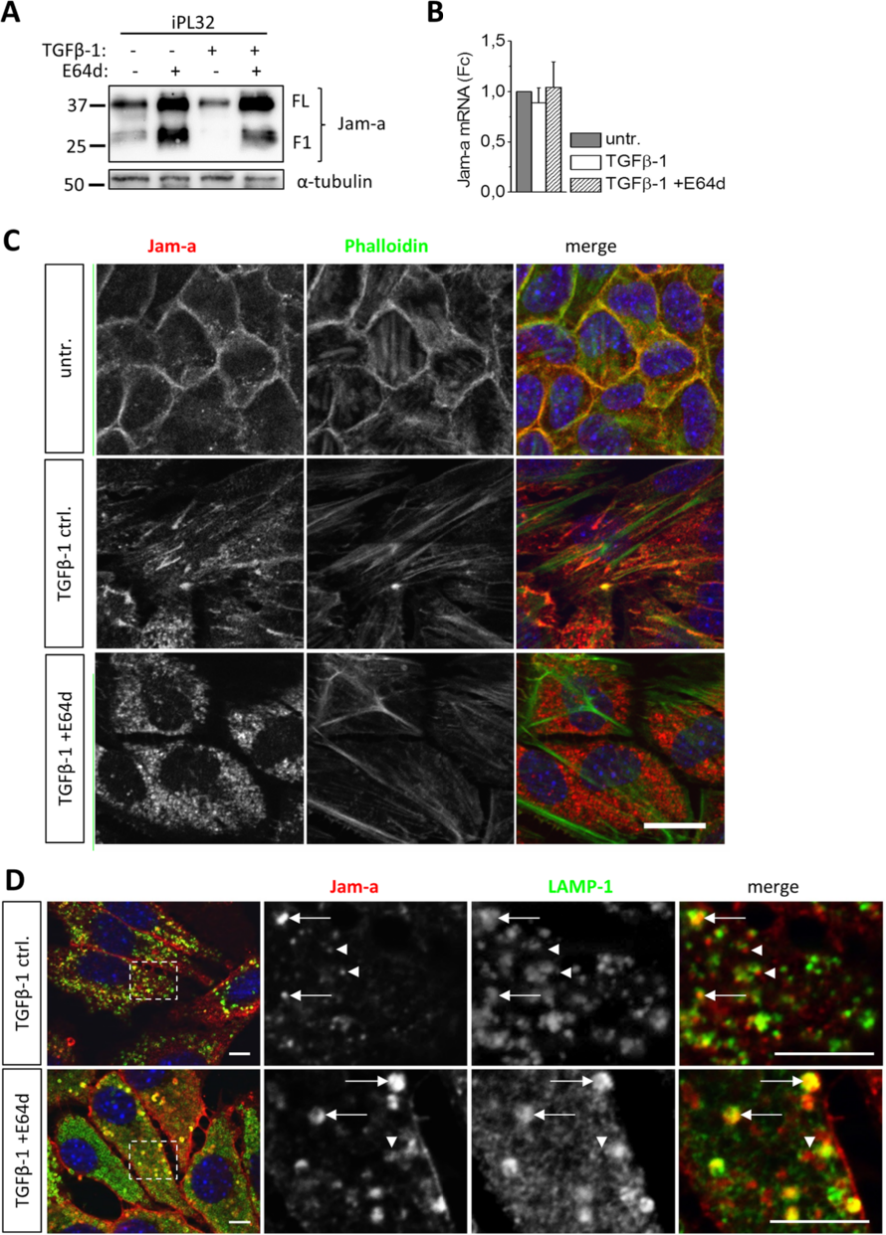
**Jam-a expression and lysosomal Jam-a accumulation upon cysteine cathepsin inhibition. (A)** Western Blot of Jam-a in whole cell lysates of untreated −/+E64d and four days TGFβ-1 −/+E64d treated iPL32 cells showed full length (FL) Jam-a and a 25 kDa Jam-a fragment (F1). **(B)** Jam-a transcription was analyzed by qRT-PCR in untreated and four days TGFβ-1 −/+E64d treated iPL32 cells. Starting quantity values were normalized to the untreated group and are shown as the mean ± SEM (n = 3). **(C)** Representative confocal images of FITC-Phalloidin (green) and Jam-a (red) immunofluorescence staining of untreated and four days TGFβ-1 −/+E64d treated iPL32 cells are shown. Images represent one confocal section at a medial position in the cells. **(D)** Representative images of optical sections of Jam-a/Lamp-1 immunofluorescence double-staining of four days TGFβ-1 −/+E64d treated iPL32 cells: Jam-a (red), Lamp-1 (green), and nuclei (blue) are shown. Dashed lines mark areas shown in higher magnification in the second, third, and fourth panels. Arrows denote Jam-a co-localization with Lamp-1 positive lysosomes. Arrowheads indicate Jam-a staining adjacent to Lamp-1 positive lysosomes. Scale bars = 10 μm.

In the context of EMT the most interesting accumulating protein identified was Junctional adhesion molecule a (Jam-a). Jam-a (or Jam-1/F11r) facilitates epithelial cell-cell adhesion and is associated with tight junctions, which establish epithelial apical-basolateral polarity. Western blot revealed not only higher full length Jam-a levels in lysates of TGFβ-1 + E64d treated iPL32 cells, but also an additional 25 kDa Jam-a fragment (Figure 7A). Jam-a transcription was not altered (Figure 7B). Immunofluorescence analysis showed Jam-a localization to lateral cell-cell contacts in untreated iPL32 cells (Figure 7C). TGFβ-1 treatment changed Jam-a subcellular localization together with a rearrangement of actin fibers. A punctate Jam-a staining pattern was observed in cells which had lost intercellular contacts. In TGFβ-1 + E64d treated cells, Jam-a massively accumulated in vesicular structures (Figure 7C). Immunofluorescence double-staining of Jam-a together with the lysosomal membrane protein Lamp-1 revealed intracellular Jam-a co-localization with Lamp-1 positive vesicles or Jam-a localization adjacent to such vesicles in TGFβ-1 treated cells (Figure 7D). The occurrence of large Jam-a/Lamp-1 double positive vesicles in TGFβ-1 + E64d treated cells confirmed lysosomal Jam-a accumulation upon cysteine cathepsin inhibition. Notably, in the non-malignant NMuMG cells accumulation of Jam-a upon cysteine cathepsin inhibition was not as pronounced as in iPL32 cancer cells (Additional file 6: Figure S3).

## Discussion

We delineate here a novel role of lysosomal cysteine cathepsin activity during TGFβ-1 induced EMT of mammary cancer cells which were established by continuous propagation of primary cancer cells derived trom the MMTV-PyMT mouse model for metastasizing breast cancers. We demonstrate that cysteine cathepsin activity contributes to invasion of the TGFβ-1 treated breast cancer cells, but not of NMuMG cells, in two ways: Firstly, the proteases support the acquisition of a TGFβ-1 induced invasive cell phenotype and secondly directly contribute to invasion of TGFβ-1 transformed cells. Our results are in concordance with the identification of Ctsb and Ctsl as mediators of the ErB2 induced invasive phenotype of MCF7 cells [24] and a multitude of experimental evidence for ECM degradation by cysteine cathepsins [15]. However, the finding that cathepsin activity was needed for acquisition of invasive cell properties during TGFβ-1 induced EMT was surprising and novel, especially since the transcriptional EMT program was not influenced by cathepsin inhibition. One should note that this effect was maintained even though time for recovery of cathepsin activities was allowed before the invasion assays. This indicates a structural change inside cells conserving the effect of the cathepsin inhibitor independent of the actual protease activity during the invasion assay.

It is known that cells regulate biogenesis and function of lysosomes in adaptation to environmental cues in order to maintain cell homeostasis [25,26]. In addition to the abundance changes of proteins from known EMT-related pathways in response to TGFβ-1, we discovered changes highlighting increased lysosome activity, including the increase of active cathepsins, increase of acidic Lamp-1 positive vesicles, and higher abundance of lysosomal membrane proteins and lysosomal enzymes. As a result of cysteine cathepsin inhibition a massive accumulation of enlarged lysosomes, reminiscent of lysosomal storage disorders, was shown. Similarly, accumulation of dysmorphic lysosomes upon loss of cysteine cathepsin activity has been observed in rat brain [27] and alveolar epithelial cells [28] and in different tissues of cathepsin L knockout mice [29-32]. Progressive accumulation of undigested material in lysosomal storage disorders results in dysfunction and cell death, especially in post-mitotic and long lived cells [33]. Here, despite the massive accumulation of large lysosomes, E64d treatment had no effect on iPL32 cell proliferation and viability, but significantly reduced invasiveness. Hence, during TGFβ-1 induced EMT lysosomal protein turnover is not essential for survival of iPL32 cells, but is important for the acquisition of an invasive cell phenotype. It is conceivable that E64d might affect cell fitness under starvation or hypoxia, because lysosomal degradation is the limiting step for the retrieval of matter and energy by autophagy. However, it is a matter of debate to which extent tumor cells depend on autophagy [6,34].

The differential role of cathepsins in NMuMG epithelial cells and iPL32 breast cancer cells might be explained by the different modes of EMT in those cell lines. In comparison to the induction of a physiological EMT in NMuMG cells upon TGFβ-1 treatment, iPL32 cells showed only a partial EMT including loss of cell-cell contacts and apical-basolateral polarity, acquisition of mesenchymal morphology, upregulation of snail and N-cadherin and increased migration and invasion without downregulation and loss of E-cadherin. It is known that depending on the origin, differentiation status and degree of malignancy, different EMT phenotypes and intermediate EMT states of mammary epithelial cells exist [35,36].

To decipher the contribution of cysteine cathepsins to TGFβ-1 induced EMT, the Jam-a accumulation in iPL32 cells found by our SILAC-proteomic approach was investigated in more detail. We provide evidence for lysosomal Jam-a accumulation as a result of cysteine cathepsin inhibition during EMT in these cells. In epithelial cells Jam-a is mostly associated with tight junctions [37]. There it facilitates cell-cell adhesion and can regulate integrinβ-1 levels, cell-matrix adhesion, as well as cell migration [38,39]. In neutrophils Jam-a concentrates at basal adhesions during directed migration where it modulates integrin β-1 internalization [40] The role of Jam-a in cancer remains ambiguous [41]. Loss of Jam-a [42-44] as well as gain of Jam-a [45,46] were reported to promote epithelial cancer cell motility. We found that TGFβ-1 caused Jam-a subcellular redistribution during EMT, while Jam-a transcription was not changed. It appears that Jam-a localization has an impact on its function. It was shown previously that loss of Ctsl changed the dynamics of the endosome/lysosome vesicular network, thereby affecting growth factor receptor trafficking and function [19,47,48]. Thus, it is possible that impaired lysosomal proteolysis also affects protein trafficking and functions during EMT. In turn this could have consequences for cell invasion. For Jam-a further characterization of its trafficking and contribution to cell invasion and metastasis is required to confirm this hypothesis. The proteome dataset presented here provides other interesting metastasis-related candidates, for example lactadherin [49] and nicastrin [50] with altered abundance upon cysteine cathepsin inhibition.

## Conclusions

Our results show an important role of lysosomes and lysosomal proteolysis in cellular remodeling during TGFβ-1 induced EMT and the acquisition of an invasive phenotype of breast cancer cells. The pivotal role of cysteine cathepsins in this process provides an additional rationale for attempts to use cathepsin inhibiting drugs to stall tumor progression and metastasis.

## Materials and methods

### Cell lines and cell culture

A subclone of NMuMG (NMuMG/E9, hereafter NMuMG) has been previously described [21] and was provided by Prof. Gerhard Christofori (University of Basel, Switzerland). A549 non-small cell lung carcinoma cells were provided by Dr. Meike Burger (University Hospital Freiburg, Germany). For the generation of the iPL32 cell line primary tumor cells were isolated from a 14 week old female FVB MMTV-PyMT luciferase transgenic mouse, as described previously [14]. Cells were cultured in DMEM supplemented with 10% fetal calf serum, 1% L-glutamine and 1% penicillin/streptomycin on plastic cell culture dishes in a sterile incubator (37°C, 5%CO_2_). When cells had grown to sub-confluence they were incubated with 0.05% trypsin-EDTA for 2 min and the dissociated cells were discarded (selective trypsinization). Remaining epithelial cells were again trypsinized until they dissociated from the culture dish, washed with DPBS and passaged. After 10 passages cells of the resulting cell line were viably frozen in culture medium containing 10% DMSO in cryovials at −80°C, stored in a liquid nitrogen tank and revitalized for experiments.

### Growth factor and inhibitor treatment

For experimental induction of EMT, NMuMG and iPL32 cells were seeded at a low density. After several cell divisions and the formation of epithelial cell clusters after two to four days, growth medium was exchanged and supplemented with human recombinant TGFβ-1 (2 ng/ml, R&D Systems). TGFβ-1 supplemented medium was exchanged every second day. A549 cells were cultured in growth medium containing 0.5% FCS during TGFβ-1 treatment. For long term cysteine cathepsin inhibition E64d (10 μM, Bachem) or DMSO as solvent control were added to the growth medium every 24 h in parallel to growth factor stimulation. For short term inhibition during the migration and invasion experiments E64d (10 μM) or DMSO were added to the growth medium one hour prior to cell dissociation and again to the cell suspension that was added to the CIM plates.

### Cell viability and proliferation

To determine cell viability 1.5×10^4^ cells were seeded in 9.6 cm^2^ wells. After two days cells were washed three times with DPBS and switched to indicator free growth medium containing 5% FCS supplemented with or without TGFβ-1+/−E64d in triplicates. Cell viability was assayed by LDH activity using the Cytotox 96® cytotoxicity assay (Promega) according to the manufacturer’s instructions. To compile growth curves, every two days cells of three wells of one condition were pooled, diluted in Trypan blue stain and living cells were counted using a Neubauer counting chamber.

### Real time cell migration and invasion

Real time cell migration and invasion through membrane-pores were determined using Cell Invasion and Migration (CIM) plates and the xCelligence® System (Roche). To analyze invasion the upper side of the membrane was coated with Cultrex® (Trevigen) diluted in serum-free growth medium to a final concentration of 680 μg/ml. After trypsin dissociation 4×10^4^ cells in 100 μl serum-free medium per well were added to the upper chamber of a CIM plate. For the following 24 h directed migration/invasion towards the lower chamber containing medium supplemented with 10% FCS, or no FCS as control, was measured in 15 min intervals.

### Quantitative RT-PCR

Total RNA from cells was extracted using the RNA mini kit (Qiagen) and 1 μg RNA/sample reverse transcribed with the iScript cDNA synthesis kit (Biorad). For quantification of gene expression Platinum CyBR® Green qPCR Super Mix (Invitrogen) and the following forward (fw) and reverse (rev) primers were used: actin: ACC CAG GCA TTG CTG ACA GG (fw) GGA CAG TGA GGC CAG GAT GG (rev); E-cadherin: GTC TAC CAA AGT GAC GCT GAA G (fw) CGG TGA TGC TGT AGA AAA CCT T (rev); Cathepsin B (Ctsb): CCT GGG CTG GGG AGT AGA GAA TGG AG (fw), TGG AAA AAG CCC CTA AGG ACT GGA CAA T (rev); Cathepsin D (Ctsd): GTG CAC ATG GAC CAG TTG GA (fw), CAA TAG CCT CAC AGC CTC CCT (rev); Cathepsin L (Ctsl): GAA TCC TAC ACT CAT CCT TGC TGC C (fw), ACA CTG CTC TCC TCC ATC CTT CTT C (rev); Jam-a: GGG CTG GAA CCT GTA GCA CC (fw), GCC AGA TCC GCG TCT ACA GC (rev); Lamp-1: GTG ACA GGT TTG GGT CTG TGG A (fw), GGT CTG ATA GCC GGC GTG AC (rev); Psmb4: ACT CGG ACC CAG AAC CCC AT (fw), CCG AGG CAC CCA GCA TAG TG (rev); Snail1: CCT TCC TCT GAC ACT TCA TCC (fw), CCT AGA CTG GGC ATC ACA GTG (rev); Zeb1: TAG CCT TAA GGA AGC AGC CA (fw), TTA AGG CCA AAG GGA CAC AG (rev).

### Western blot

Whole cell protein extracts were prepared by on plate cell lysis with TritonX-100 buffer (50 mM Tris pH 7.5, 150 mM NaCl, 0.5% Triton X-100) or RIPA buffer (50 mM Tris HCL (pH 8.0), 250 mM NaCl, 2% NP-40, 0.5% sodium-deoxycholate, 0.7% SDS, 2.5 mM EDTA) containing 40 μl/ml stock solution (1 tablet/2 ml lysis buffer) cOmplete protease inhibitor cocktail (Roche). Cell lysates were frozen at −80 C. After thawing on ice cells were homogenized by passing through an Ø 0.45 mm syringe 10 times and centrifuged for 5 min at 1000rcf at 4°C. The supernatant was collected and stored at −80°C or directly used for analysis. Protein concentrations were determined with bisinchoninic acid protein assay (BCA, Pierce). Equal amounts of protein extracts (20 μg or 40 μg) were resolved by SDS-PAGE and transferred onto polyvinylidene fluoride (PVDF) membranes using a semi-dry system (Biorad). For immune detection of proteins, membranes were probed with the following antibody dilutions: primary: actin: MP 69100 (1: 2000); Ctsb: R&D systems AF965 (1:250); Ctsc R&D Systems AF1034 (1:50); Ctsd R&D systems AF1029 (1:200); Ctsl: R&D systems AF1515 (1:500); Ctsh R&D Systems AF 1013 (1: 250); Ctsz biotin. R&D systems BAF1033 (1:500); E-cadherin: BD Bioscience 610182 (1:500); Jam-a: R&D systems AF1077 (1:500); Lamp-1: Abcam ab25245 (1:500); N-cadherin: Cell signalling #4061 (1:100); tubulin: Sigma #T9026 (1:1000); secondary: anti-mouse HRP: Sigma AO168 (1:5000); anti-rat HRP: Jackson #112-035-062 (1:5000); anti-goat HRP: Sigma A5420 (1:5000) or streptavidin-HRP: Roche (1:5000). HRP activity was detected using West Pico or Femto chemiluminescent substrate (Pierce) and Fusion SL Imager (Vilber Lourmant).

### Cysteine cathepsin and proteasome activity

Protein extracts were prepared as described for Western blot, except cells were lysed in reducing sodium acetate buffer (100 mM sodium acetate, 1 mM EDTA, 0.05% Brij, pH 5.5) to measure cathepsin activity and in proteasome assay buffer (50 mM Tris, 25 mM KCl, 10 mM NaCl, 1 mM MgCl_2_, 0.5 mM DTT, 0,05% NP-40, pH 7.5) to measure proteasome activity. Ctsb and Ctsl proteolytic activity were determined as described previously [51]. Ctsl activity was defined as zPheArg-AMC hydrolysis (without the cathepsin B inhibitor Ca074) - zPheArg-AMC hydrolysis (with Ca074). Proteasome activity was measured using the 20S Proteasome Activity Assay Kit (Millipore). Enzyme activities were calculated by the slopes in the linear regions of curves, showing RFU over time, relative to the slopes of AMC standard curves.

### Flow cytometry

Cells were washed with DPBS and incubated with 0.1 μM LysoTracker™ Green (Molecular Probes) or 1 μg/ml Acridine-Orange in FACS buffer (DPBS +3% fetal calf serum) for 15 min at 37°C in a CO_2_ incubator. Subsequently cells were washed twice with DPBS, trypsinized, washed again with DPBS and resuspended in FACS buffer. Green fluorescence (FL-1) of LysoTracker™ Green stained cells as well as green (FL-1) and orange (FL-3) fluorescence of Acridine-Orange stained cells were analyzed by flow cytometry (FACScalibur, Fortessa) and evaluated with FlowJo, At least 50.000 events were analyzed. Dead cells were excluded from the analysis by size.

### Immunofluorescence and microscopy

Cells were cultivated on cover glass slides in 24-well culture plates. After washing with PBS four times cells were fixed in 4% PFA for 30 min. at RT. All following washing and incubation steps were carried out with PBST on a rocker: Cells were washed for 5 min four times and permeabilized with 0.05% saponin in PIPES for 5 min at RT. After another three washing steps, cells were fixed with pre-chilled ice cold 100% acetone for 2 min on ice and immediately washed three times. Blocking with 4% BSA in PBST for 30 min at RT was followed by overnight incubation at 4°C with the primary antibody. The next day cells were washed three times and incubated with the secondary antibody for 1 h at RT. For double-staining the procedure was repeated with the next antibodies. After repeated PBST and PBS washing steps, slides were mounted with Permafluor®. The following antibodies were used: Lamp-1 Abcam ab25245 (1:700); anti-rat Alexa fluor 488 Invitrogen A11006 (1:1500); Jam-a R&D systems AF1077 (1:50); anti-goat Alexa fluor 594 Invitrogen A11080 (1:1500). To show changes in cell shape, actin filaments were visualized with FITC-Phalloidin (Millipore). Cells were imaged with a fluorescent Microscope (Axiovert 40C, Zeiss). For optical sections the ApoTome.2 (Zeiss) was used with the Axiovert 40C microscope or cells were imaged with a Leica TCS SP2 AOBS confocal microscope.

### Quantitative proteome comparison

For stable isotopic labeling with amino acids in culture (SILAC) cells were cultivated in DMEM (minus L-arg and L-lys, Silantes) supplemented with 10% dialyzed FCS and glutamine containing either L-arginine (Arg0) and L-lysine (Lys0) “light” or ^13^C_6_ L-arginine (Arg6) and ^2^H_4_ L-lysine (Lys4) “medium” or ^13^C_6_^15^ N_4_ L-arginine (Arg10) and ^13^C_6_^15^ N_2_ L-lysine (Lys8) “heavy” for at least two weeks (five passages). For each experiment 2x10^5^ pre-labeled cells/10 cm culture dish were seeded. Light labeled cells were cultured without TGFβ-1 (untreated), medium labeled cells with TGFβ-1 and heavy labeled cells with TGFβ-1 + E64d in two replicates per condition for four days in the respective SILAC media. Cells were lysed on plates with 400 μl lysis buffer (50 mM Tris pH 7.5, 150 mM NaCl, 1% Triton X-100, 1% sodium deoxycholate, 0.02% SDS, 1 mM EDTA). Cell lysates of replica plates were combined and centrifuged at 1000 g for 5 min at 4°C and the supernatant was used. Protein concentrations were determined by BCA. Equal protein amounts of light, medium and heavy labeled samples were combined. The volume of the combined lysates was concentrated using Amnicon® Ultra 2 ml centrifugal filters (Milipore) and Vivaspin 500 10 kDa cut-off spin filters (Satorius). Protein mixtures were separated by SDS-PAGE (4% - 12% gradient Criterion Tris HCL gel, biorad and NuPage® buffers, Invitrogen) and in-gel digested with sequencing grade trypsin (Promega). The resulting peptide mixtures were desalted using self- packed C18 STAGE tips (Empore) [52]. For nanoflow-LC–MS/MS, samples were analyzed on an Orbitrap XL (Thermo Scientific) mass spectrometer. The instrument was coupled to an Ultimate3000 micro pump (Thermo Scientific) with a flow rate of 300 nl/min; 0.5% acetic acid and 0.5% acetic acid in 80% acetonitrile (water and acetonitrile were at least HPLC gradient grade quality) with a gradient of increasing organic proportion were used for peptide separation. Column tips with 75-μm inner diameter and 11-cm length were self-packed with Reprosil-Pur 120 ODS-3 (Dr. Maisch). The mass spectrometer was operated in the data-dependent mode and switched automatically between MS and MS/MS. Raw mass spectrometric data was analyzed with MaxQuant Version 1.3.0.5. A peptide confidence level at 95% and a protein false discovery rate of <1.0% were chosen. The precursor mass tolerance was set to 6 ppm. A fragment mass tolerance of 0.5 Da was chosen. No variable modifications and the following static modifications were set: Lys0/Arg0 (light), Lys4/Arg6 (medium), Lys8/Arg10 (heavy). The data was compared to the complete UniProt mouse database consisting of 44819 protein entries (downloaded on October 16th, 2011). Randomized decoy sequences were additionally generated by the MaxQuant algorithm.

### Statistical analysis

Statistical analyses were performed with Origin® (Microcal) employing two sample two sided t-tests for the comparison of two groups, or one sample two sided t-tests for the comparison of one group normalized to another group. All values represent mean ± SEM of at least three independent experiments; *p ≤ 0.05, **p ≤ 0.01, ***p ≤ 0.001.

## Additional files

Additional file 1: Figure S1. Contribution of lysosomal proteolysis to TGFβ-1 induced invasion of A549 non-small cell lung carcinoma cells. (A) Representative phase contrast images show untreated and four days TGFβ-1 −/+E64d treated A549 cells. (B) Acidic organelles of untreated and four days TGFβ-1 treated A549 cells were analyzed by quantitative LysoTracker™ flow cytometry and are shown as the mean ± SEM (n = 3, ***p ≤ 0.05 by two tailed t-test for independent samples). (C) Migration and invasion of A549 cells: Graphs show the cell indexes of triplicates during the time course of representative experiments. Bar graphs show the statistical analysis of independent experiments calculated as the slope of cell index between the time points marked in the time-curves, normalized to “TGFβ-1 ctrl”. Data are shown as the mean ± SEM (n = 4). (D) Flow cytometry of Acridine-Orange (AO) stained A549 cells pretreated with or without TGFβ-1 −/+E64d for four days: Representative histograms for FL-3 height (orange) and FL-1 height (green) and statistical analysis of independent experiments are shown. Geometric mean orange or green fluorescence was normalized to untreated control cells (n = 3, *p ≤ 0.05). Additional file 2: Figure S2. EMT marker gene expression, cell viability, and cell proliferation were not affected by cysteine cathepsin inhibition. (A) Representative phase contrast images show morphological changes in NMuMG and iPL32 cells after two and four days TGFβ-1 −−/+E64d (10 μM) treatment. (B,C) qRT-PCR analysis of snail1, zeb1 and E-cadherin transcription relative to β-actin in (B) NMuMG and (C) iPL32 cells after two and four days TGFβ-1 and TGFβ-1 + E64d treatment. Starting quantity values were normalized to the untreated group (d0). Data are shown as the mean ± SEM (n = 3, *p ≤ 0.05 **p ≤ 0.01). (D) Western Blot of E-cadherin (E-cad) and N-cadherin (N-cad) in iPL32 whole cell lysates after four days −/+TGFβ-1 −/+E64d treatment with actin as loading control. (E) The effect of two days of TGFβ-1 −/+E64d treatment on NMuMG and iPL32 cell viability measured by lactate dehydrogenase “LDH” release was analyzed (% of total cellular LDH, n = 3, *p ≤ 0.05 **p ≤ 0.01 by two tailed t-test for independent samples). (F) Number of living untreated and TGFβ-1 treated NMuMG and iPL32 cells after two, four, and six days E64d treatment was counted by trypan blue exclusion with a Neubauer cell counting chamber (n = 3). Additional file 3: Table S1. List of all proteins with lower abundance upon TGFβ-1 treatment of iPL32 cells identified in the quantitative proteome comparison: All proteins with log2 fold change (FC) ratios TGFβ-1/untreated ≤ − 0.58 (highlighted in grey) in both experiment 1 “E1” and experiment 2 “E2” are shown with proteins most altered on top. The respective log2 FC ratios TGFβ-1/TGFβ-1 + E64d are also shown. Additional file 4: Table S2. List of all proteins with higher abundance upon TGFβ-1 treatment of iPL32 identified in the quantitative proteome comparison: All proteins with log2 fold change (Fc) ratios TGFβ-1/untreated ≥ 0.58 (highlighted in grey) in both experiment1 “E1” and experiment2 “E2” are shown with proteins most altered on top. The respective log2 FC ratios TGFβ-1/TGFβ-1 + E64d are also shown. Lysosomal proteins as determined by KEGG are highlighted in blue. Additional file 5: Table S3. List of proteins with altered abundance upon cysteine cathepsin inhibition in TGFβ-1 treated iPL32 cells identified in the quantitative proteome comparison: All proteins at least 50% higher or lower upon E64d treatment (log2 Fc TGFβ-1/untr. ≤ −0.58 or ≥ 0.58, highlighted in grey) in both experiment 1 “E1” and experiment 2 “E2” are shown. The respective log2 FC ratios TGFβ-1/untr. are also shown. Additional file 6: Figure S3. Jam-a expression in NMuMG cells. (A) Protein levels of Jam-a in whole cell lysates of untreated −/+E64d and four days TGFβ-1 −/+E64d treated NMuMG and iPL32 cells were compared by Western blot. (B) Representative confocal microscopy images of FITC-Phalloidin (green) and Jam-a (red) immune fluorescence staining of NMuMG cells that were either untreated or TGFβ-1 −/+E64d treated for four days are shown. Scale bar = 10 μm. Images represent one confocal section at a medial position in the cells.

## Abbreviations

Ctsb: Cathepsin B
Ctsl: Cathepsin L
EMT: Epithelial-to-mesenchymal transition
iPL32: Immortalized polyoma-luciferase 32
Jam-a: Junctional adhesion molecule A
Lamp: Lysosomal associated membrane protein
MMTV-PyMT: Mouse mammary tumor virus – polyoma middle T
NMuMG: Normal murine mammary gland
SILAC: Stable isotopic labeling with amino acids in culture
TGFβ: Transforming growth factor beta
