# Supplementary figures and images for: Lysosomal protein turnover contributes to the acquisition of TGFβ-1 induced invasive properties of mammary cancer cells
Source: Mol Cancer. 2015 Feb 15;14:39. doi: 10.1186/s12943-015-0313-5 (PMC4339013; doi:10.1186/s12943-015-0313-5)

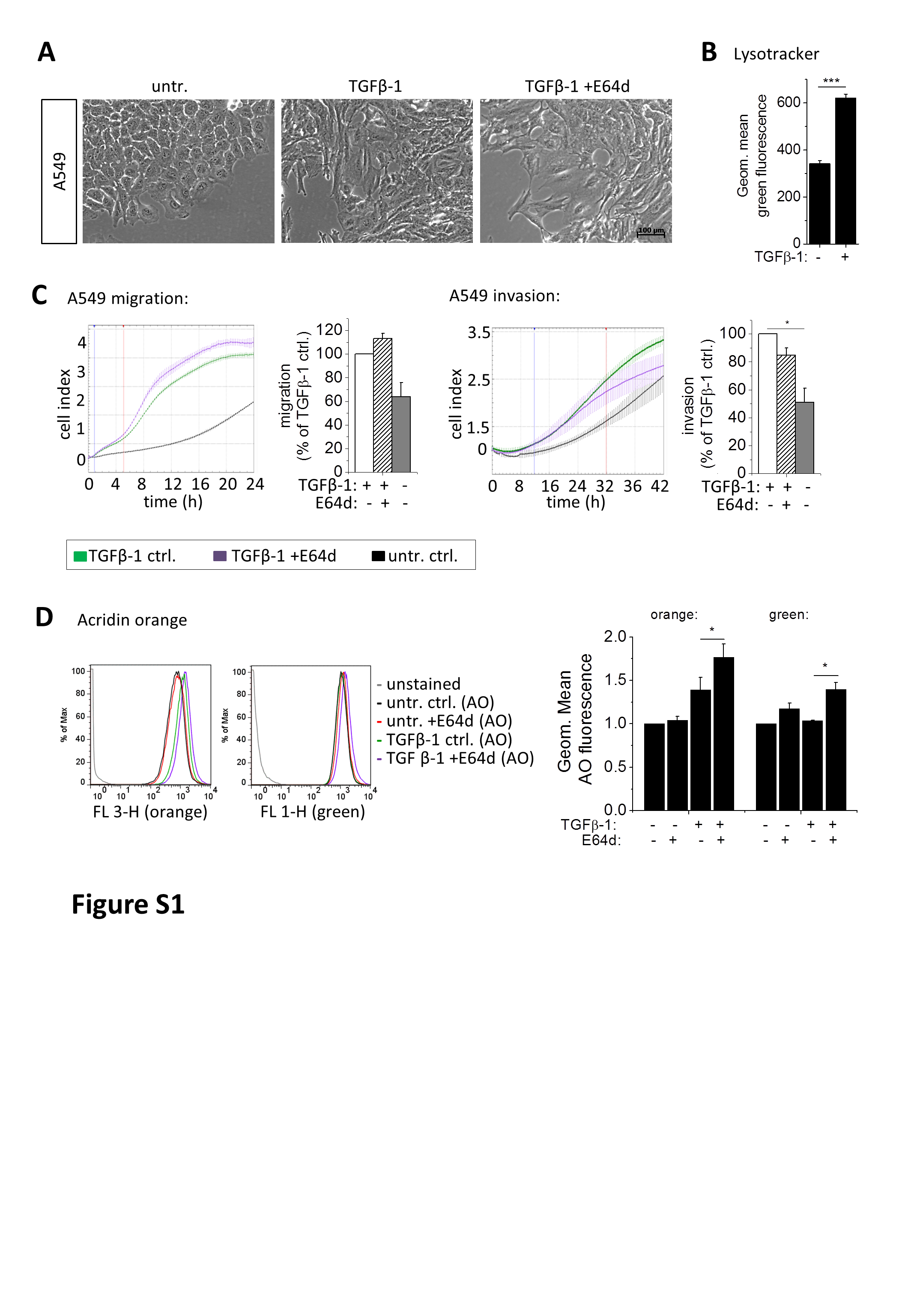

Supplement: Additional file 1: Figure S1. — Contribution of lysosomal proteolysis to TGFβ-1 induced invasion of A549 non-small cell lung carcinoma cells. (A) Representative phase contrast images show untreated and four days TGFβ-1 −/+E64d treated A549 cells. (B) Acidic organelles of untreated and four days TGFβ-1 treated A549 cells were analyzed by quantitative LysoTracker™ flow cytometry and are shown as the mean ± SEM (n = 3, ***p ≤ 0.05 by two tailed t-test for independent samples). (C) Migration and invasion of A549 cells: Graphs show the cell indexes of triplicates during the time course of representative experiments. Bar graphs show the statistical analysis of independent experiments calculated as the slope of cell index between the time points marked in the time-curves, normalized to “TGFβ-1 ctrl”. Data are shown as the mean ± SEM (n = 4). (D) Flow cytometry of Acridine-Orange (AO) stained A549 cells pretreated with or without TGFβ-1 −/+E64d for four days: Representative histograms for FL-3 height (orange) and FL-1 height (green) and statistical analysis of independent experiments are shown. Geometric mean orange or green fluorescence was normalized to untreated control cells (n = 3, *p ≤ 0.05). [file 12943_2015_313_MOESM1_ESM.tiff]

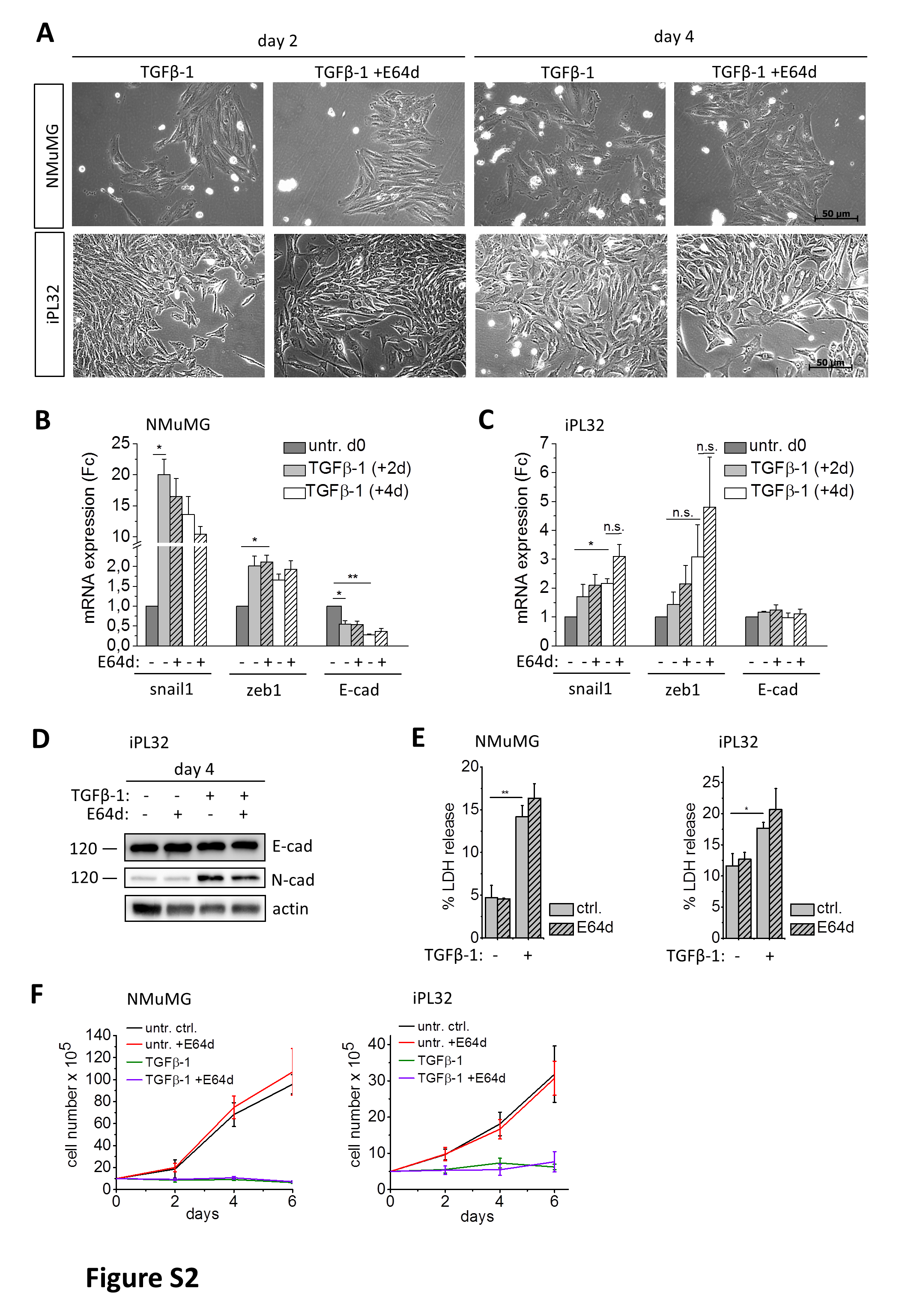

Supplement: Additional file 2: Figure S2. — EMT marker gene expression, cell viability, and cell proliferation were not affected by cysteine cathepsin inhibition. (A) Representative phase contrast images show morphological changes in NMuMG and iPL32 cells after two and four days TGFβ-1 −−/+E64d (10 μM) treatment. (B,C) qRT-PCR analysis of snail1, zeb1 and E-cadherin transcription relative to β-actin in (B) NMuMG and (C) iPL32 cells after two and four days TGFβ-1 and TGFβ-1 + E64d treatment. Starting quantity values were normalized to the untreated group (d0). Data are shown as the mean ± SEM (n = 3, *p ≤ 0.05 **p ≤ 0.01). (D) Western Blot of E-cadherin (E-cad) and N-cadherin (N-cad) in iPL32 whole cell lysates after four days −/+TGFβ-1 −/+E64d treatment with actin as loading control. (E) The effect of two days of TGFβ-1 −/+E64d treatment on NMuMG and iPL32 cell viability measured by lactate dehydrogenase “LDH” release was analyzed (% of total cellular LDH, n = 3, *p ≤ 0.05 **p ≤ 0.01 by two tailed t-test for independent samples). (F) Number of living untreated and TGFβ-1 treated NMuMG and iPL32 cells after two, four, and six days E64d treatment was counted by trypan blue exclusion with a Neubauer cell counting chamber (n = 3). [file 12943_2015_313_MOESM2_ESM.tiff]

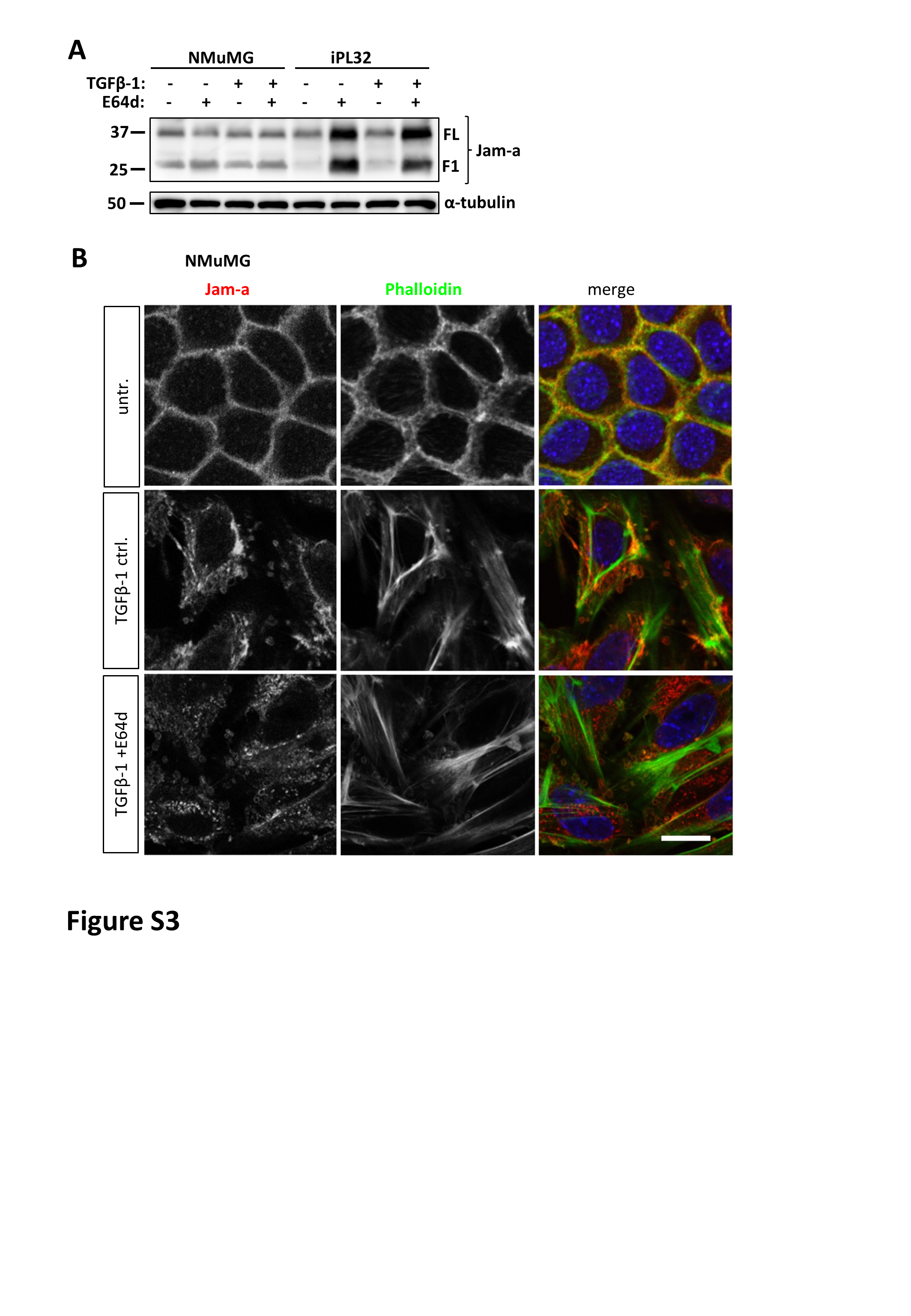

Supplement: Additional file 6: Figure S3. — Jam-a expression in NMuMG cells. (A) Protein levels of Jam-a in whole cell lysates of untreated −/+E64d and four days TGFβ-1 −/+E64d treated NMuMG and iPL32 cells were compared by Western blot. (B) Representative confocal microscopy images of FITC-Phalloidin (green) and Jam-a (red) immune fluorescence staining of NMuMG cells that were either untreated or TGFβ-1 −/+E64d treated for four days are shown. Scale bar = 10 μm. Images represent one confocal section at a medial position in the cells. [file 12943_2015_313_MOESM6_ESM.tiff]
