# Supplementary material for: Lysosomal protein turnover contributes to the acquisition of TGFβ-1 induced invasive properties of mammary cancer cells
Source: Mol Cancer. 2015 Feb 15;14:39. doi: 10.1186/s12943-015-0313-5 (PMC4339013; doi:10.1186/s12943-015-0313-5)
Supplement: Additional file 3: Table S1. — List of all proteins with lower abundance upon TGFβ-1 treatment of iPL32 cells identified in the quantitative proteome comparison: All proteins with log2 fold change (FC) ratios TGFβ-1/untreated ≤ − 0.58 (highlighted in grey) in both experiment 1 “E1” and experiment 2 “E2” are shown with proteins most altered on top. The respective log2 FC ratios TGFβ-1/TGFβ-1 + E64d are also shown. [file 12943_2015_313_MOESM3_ESM.pdf]

- Supplementary Information Table S1 -

Proteins less abundant upon TGFβ-1 treatment

Protein name	Protein ID	Gene name	E1		E2	
			log2FC (TGFβ-1/untr.)	log2FC (TGFβ-1 /TGFβ-1+E64d))	log2FC (TGFβ-1/untr.)	log2FC (TGFβ-1 /TGFβ-1+E64d))
Sodium-coupled monocarboxylate transporter 1	Q8BYF6	Slc5a8	-2,75	0,44	-2,67	0,46
Interferon-induced very large GTPase 1	F8WJ19	Gvin1	-2,73	-0,28	-2,20	-0,38
Alpha-2-macroglobulin	D3YW52	Pzp	-2,26	-0,35	-2,81	0,30
Interleukin-1 receptor antagonist protein	P25085	Il1rn	-2,12	-0,10	-2,93	0,15
Structural maintenance of chromosomes protein	E9Q1E9	Smc2	-2,02	0,15	-1,88	1,27
DNA replication licensing factor MCM4	P49717	Mcm4	-1,96	0,28	-0,85	0,33
Cyclin-dependent kinase 1	P11440	Cdk1	-1,87	-0,35	-1,96	0,37
DNA replication licensing factor MCM6	P97311	Mcm6	-1,73	0,06	-1,53	-0,05
DNA replication licensing factor MCM2	P97310	Mcm2	-1,68	-0,04	-1,86	-0,01
High mobility group protein B2	P30681	Hmgb2	-1,66	-0,01	-1,56	-0,20
DNA replication licensing factor MCM7	Q61881	Mcm7	-1,64	0,02	-2,24	-0,19
Proliferating cell nuclear antigen	P17918	Pcna	-1,62	-0,10	-1,52	0,34
DNA (cytosine-5)-methyltransferase 1	P13864	Dnmt1	-1,45	-0,10	-1,49	0,43
Stathmin	P54227	Stmn1	-1,41	0,18	-1,83	0,17
Periplakin	Q9R269	Ppl	-1,41	0,16	-1,63	0,00
Vacuolar protein sorting-associated protein 28 homolog	Q9D1C8	Vps28	-1,40	1,10	-1,22	-0,29
Galectin-3	P16110	Lgals3	-1,38	0,84	-2,29	1,68
Acidic leucine-rich nuclear phosphoprotein 32 family member E	P97822	Anp32e	-1,31	0,15	-1,20	0,03
Claudin-7	Q9Z261	Cldn7	-1,26	0,53	-0,74	-0,12
Metalloproteinase inhibitor 3	P39876	Timp3	-1,26	0,26	-1,09	0,70
Plasminogen activator inhibitor 2, macrophage	P12388	Serpinb2	-1,21	-0,01	-1,62	0,43
UPF0160 protein MYG1, mitochondrial	Q9JK81	Myg1	-1,21	0,27	-1,07	0,15
Epiplakin	Q8R0W0	Eppk1	-1,20	-0,03	-1,54	0,16
Small subunit processome component 20 homolog	E9QK83	Utp20	-1,18	-0,10	-1,48	0,39
Cytoplasmic aconitate hydratase	Q8VDC3	Aco1	-1,12	0,31	-1,21	0,26
Bifunctional 3-phosphoadenosine 5-phosphosulfate synthase 1	Q60967	Papss1	-1,06	0,11	-1,21	0,10
E3 ubiquitin-protein ligase RNF213	E9Q555	Rnf213	-1,06	0,29	-1,31	0,30
40S ribosomal protein S26	P62855	Rps26	-1,06	-0,37	-0,65	-0,24
Histone H1.3;Histone H1.2	P43277	Hist1h1d	-1,05	0,05	-0,67	-0,05
Protein-glutamine gamma-glutamyltransferase 2	P21981	Tgm2	-1,03	-0,13	-1,14	0,04
Glutathione S-transferase A4	P24472	Gsta4	-0,99	0,10	-1,58	0,19
60S ribosomal protein L35	Q6ZWV7	Rpl35	-0,98	-0,08	-0,71	0,08

Protein name	Protein ID	Gene name	E1 log2FC (TGFβ-1/untr.)	E1 log2FC (TGFβ-1 /TGFβ-1+E64d))	E2 log2FC (TGFβ-1/untr.)	E2 log2FC (TGFβ-1 /TGFβ-1+E64d))
Acetyl-CoA carboxylase 1	E9PUB1	Acaca	-0,98	0,38	-1,18	0,59
p21-activated protein kinase-interacting protein 1	Q9DCE5	Pak1ip1	-0,97	0,17	-0,88	-0,35
Deoxyuridine triphosphatase, isoform CRA_b	Q8VCG1	Dut	-0,95	0,03	-0,81	0,08
Aminoacyl tRNA synthase complex-interacting multifunctional protein 2	Q8R010	Aimp2	-0,91	0,42	-0,73	0,36
Acyl-CoA-binding protein	F6ZM12	Dbi	-0,91	0,25	-1,17	0,51
Ceruloplasmin	E9PZD8	Cp	-0,88	0,37	-0,88	0,41
m7GpppX diphosphatase	Q9DAR7	Dcps	-0,88	-0,10	-1,04	0,03
Proteasome assembly chaperone 3	Q9CZH3	Psmg3	-0,88	0,23	-0,89	0,25
Basic leucine zipper and W2 domain-containing protein 2	Q91VK1	Bzw2	-0,88	-0,52	-0,80	-0,08
UTP--glucose-1-phosphate uridylyltransferase	Q91ZJ5	Ugp2	-0,87	0,31	-1,39	0,73
Atp2b1 protein	Q05CJ5	Atp2b1	-0,87	0,08	-1,00	-0,04
Integrin alpha-6;Integrin alpha-6 heavy chain;Integrin alpha-6 light chain	Q61739	Itga6	-0,86	-0,06	-1,09	0,07
RRP12-like protein	Q6P5B0	Rrp12	-0,85	-0,28	-0,86	0,02
Protein diaphanous homolog 1	E9PV41	Diap1	-0,85	0,31	-0,72	0,27
Transferrin receptor protein 1	Q62351	Tfrc	-0,84	1,36	-0,85	1,84
C-1-tetrahydrofolate synthase, cytoplasmic	F6YW06	Mthfd1	-0,83	0,01	-0,88	0,09
Superkiller viralicidic activity 2-like 2	Q9CZU3	Skiv2l2	-0,82	0,02	-0,68	-0,38
Protein DEK	Q7TNV0	Dek	-0,82	-0,11	-0,90	-0,02
Delta-aminolevulinic acid dehydratase	P10518	Alad	-0,82	0,31	-0,79	0,26
Dihydrofolate reductase	P00375	Dhfr	-0,80	0,12	-1,61	0,02
Ubiquitin carboxyl-terminal hydrolase isozyme L5	Q9WUP7	Uchl5	-0,79	0,17	-0,61	0,20
Lysine-specific demethylase 3B	B9EKS2	Kdm3b	-0,77	-0,17	-0,76	-0,13
Pyruvate carboxylase	E9QPD7	Pcx;Pc	-0,76	0,17	-0,75	-0,22
Cordon-bleu protein-like 1	E9QPU9	Cobll1	-0,76	-0,10	-0,59	0,01
L-lactate dehydrogenase A chain dehydrogenase	P06151	Ldha	-0,73	0,06	-0,83	0,38
Epithelial splicing regulatory protein 1	F8WGU3	Esrp1	-0,73	-0,28	-0,62	-0,34
Sickle tail protein	E9QAU4	Etl4;Skt	-0,73	-0,09	-1,07	-0,11
General transcription factor II-I	Q9ESZ8	Gtf2i	-0,72	0,06	-0,69	-0,01
	E9Q4Z2	Acacb	-0,71	0,27	-1,04	0,67
40S ribosomal protein S15	P62843	Rps15	-0,70	-0,13	-0,86	0,08
Pre-rRNA-processing protein TSR1 homolog	Q5SWD9	Tsr1	-0,70	0,00	-1,39	0,09
Monocarboxylate transporter 1	P53986	Slc16a1	-0,70	0,00	-0,60	0,05

Protein name	Protein ID	Gene name	E1		E2	
			log2FC (TGFβ-1/untr.)	log2FC (TGFβ-1 /TGFβ-1+E64d))	log2FC (TGFβ-1/untr.)	log2FC (TGFβ-1 /TGFβ-1+E64d))
Ubiquitin-like protein 4A	P21126	Ubl4a	-0,68	0,24	-0,63	0,42
Ribosomal RNA processing protein 1 homolog A	P56183	Rrp1	-0,67	-0,14	-0,70	-0,40
Far upstream element-binding protein 1	Q91WJ8	Fubp1	-0,67	-0,05	-0,89	-0,11
Fatty acid-binding protein, epidermal	Q05816	Fabp5	-0,66	0,23	-0,71	0,28
Glyoxylate reductase/hydroxypyruvate reductase	Q91Z53	Grhpr	-0,65	0,19	-0,75	0,06
Choline dehydrogenase, mitochondrial	Q8BJ64	Chdh	-0,64	-0,01	-1,23	0,74
Perilipin-3	Q9DBG5	Plin3	-0,64	0,45	-0,73	0,23
Acidic leucine-rich nuclear phosphoprotein 32 family member B	E9QKP8	Anp32b	-0,64	-0,06	-0,77	-0,05
Microsomal glutathione S-transferase 1	E9QJW0	Mgst1	-0,63	0,24	-0,60	0,16
Aldehyde dehydrogenase	B1ATI0	Aldh3a2	-0,63	0,09	-0,83	0,24
40S ribosomal protein S27	Q6ZWU9	Rps27	-0,63	-0,43	-0,67	-0,18
D-3-phosphoglycerate dehydrogenase	Q61753	Phgdh	-0,62	0,19	-1,04	0,53
Huntingtin	P42859	Htt	-0,62	0,38	-0,64	0,67
Valine--tRNA ligase	Q9Z1Q9	Vars	-0,61	-0,03	-0,75	0,21
Sister chromatid cohesion protein PDS5 homolog B	F8WHU5	Pds5b	-0,61	-0,17	-0,87	-0,36
Treacle protein	O08784	Tcof1	-0,61	0,21	-0,62	-0,01
Adenylosuccinate synthetase isozyme 2	P46664	Adss	-0,61	-0,04	-0,60	-0,30
Heat shock protein 105 kDa	Q61699	Hsph1	-0,60	0,06	-0,69	0,28
Lamina-associated polypeptide 2, isoforms beta/delta/epsilon/gamma	Q61029	Tmpo	-0,60	0,03	-0,72	-0,03
28 kDa heat- and acid-stable phosphoprotein	Q3UHX2	Pdap1	-0,60	0,10	-0,60	-0,06
Importin-4	Q8VI75	Ipo4	-0,59	0,11	-0,66	0,15
Leucine-rich repeat-containing protein 16A	D3Z024	Lrrc16a	-0,59	-0,20	-0,59	-0,40
E3 ubiquitin-protein ligase UBR5	E9Q2H1	Ubr5	-0,59	0,05	-0,72	-0,02
Myb-binding protein 1A	Q7TPV4	Mybbp1a	-0,59	-0,21	-0,87	-0,08
Structural maintenance of chromosomes protein 1A	Q9CU62	Smc1a	-0,58	-0,15	-0,75	-0,13

Table S1: List of all proteins with lower abundance upon TGFβ-1 treatment of iPL32 cells identified in the quantitative proteome comparison: All proteins with $\log_2$ fold change (FC) ratios TGFβ-1/untreated ≤ - 0.58 (highlighted in grey) in both experiment 1 “E1”

and experiment 2 “E2” are shown with proteins most altered on top. The respective $\log_2$ FC ratios TGFβ-1/TGFβ-1+E64d are also shown.
