# Supplementary material for: Lysosomal protein turnover contributes to the acquisition of TGFβ-1 induced invasive properties of mammary cancer cells
Source: Mol Cancer. 2015 Feb 15;14:39. doi: 10.1186/s12943-015-0313-5 (PMC4339013; doi:10.1186/s12943-015-0313-5)
Supplement: Additional file 4: Table S2. — List of all proteins with higher abundance upon TGFβ-1 treatment of iPL32 identified in the quantitative proteome comparison: All proteins with log2 fold change (Fc) ratios TGFβ-1/untreated ≥ 0.58 (highlighted in grey) in both experiment1 “E1” and experiment2 “E2” are shown with proteins most altered on top. The respective log2 FC ratios TGFβ-1/TGFβ-1 + E64d are also shown. Lysosomal proteins as determined by KEGG are highlighted in blue. [file 12943_2015_313_MOESM4_ESM.pdf]

Proteins more abundant upon TGFβ-1 treatment

Protein name	Protein ID	Gene name	E1 log$_2$ FC (TGFβ-1 /untr.)	E1 log$_2$ FC (TGFβ-1 /TGFβ-1+E64d)	E2 log$_2$ FC (TGFβ-1 /untr.)	E2 log$_2$ FC (TGFβ-1 /TGFβ-1+E64d)
Collagen alpha-2(V) chain	Q3U962	Col5a2	3,61	-0,31	3,32	-0,08
Clusterin	Q06890	Clu	3,60	0,07	3,10	-0,18
Transgelin	P37804	Tagln	3,51	-0,61	2,47	0,36
Vimentin	P20152	Vim	3,43	-1,00	1,64	1,84
Collagen alpha-1(XII) chain	Q60847	Col12a1	3,04	-1,16	2,32	-0,65
Cysteine and glycine-rich protein 2	P97314	Csrp2	2,93	-0,40	2,98	-0,91
Erythrocyte band 7 integral membrane protein	P54116	Stom	2,92	0,56	0,74	0,52
Collagen alpha-1(XI) chain	Q61245	Col11a1	2,66	-0,91	1,95	-0,17
Protein Fat1	F2Z4A3	Fat1	2,53	-0,51	2,38	-0,62
Drebrin	Q9QXS6	Dbn1	2,49	-0,42	2,82	-0,56
Tenascin	Q80YX1	Tnc	2,44	-0,45	2,16	-0,11
Neural cell adhesion molecule 1	P13595	Ncam1	2,44	-0,22	2,15	-0,34
Immunity-related GTPase family M protein 1	Q60766	Irgm1	2,31	-0,51	1,25	0,18
Cytoskeleton-associated protein 4	Q8BMK4	Ckap4	2,07	-0,28	2,07	0,09
5-nucleotidase	Q61503	Nt5e	1,95	0,67	1,73	0,60
Integrin alpha-V	P43406	Itgav	1,89	0,05	1,91	-0,08
Cell surface glycoprotein MUC18	Q8R2Y2	Mcam	1,81	-0,38	1,99	-0,45
Adenylate kinase isoenzyme 1	Q9R0Y5	Ak1	1,80	0,05	1,67	0,27
Integrin beta;Integrin beta-5	Q6PE70	Itgb5	1,77	0,00	1,84	-0,60
Syndecan-1	P18828	Sdc1	1,75	-0,19	1,41	0,32
Voltage-dependent anion-selective channel protein 3	Q60931	Vdac3	1,75	0,00	1,09	-0,09
Protein Myl12a	Q6ZWQ9	Myl12a	1,74	-0,27	1,87	-0,23
Latent-transforming growth factor beta-binding protein 2	O08999	Ltbp2	1,73	-0,31	2,44	-0,75
Integrin alpha-2	Q62469	Itga2	1,54	0,32	1,97	0,15
Prolyl 4-hydroxylase subunit alpha-1	Q60715	P4ha1	1,48	-0,43	1,87	-0,34
Voltage-dependent anion-selective channel protein 2	Q60930	Vdac2	1,47	0,16	1,07	0,06
Protein Ahnak2	E9PYB0	Ahnak2	1,47	0,36	1,73	0,50
14-3-3 protein sigma	O70456	Sfn	1,46	-0,09	0,94	0,37
Creatine kinase B-type	Q04447	Ckb	1,45	-0,23	1,82	-0,69
Cytochrome c oxidase subunit 7A2, mitochondrial	P48771	Cox7a2	1,42	-0,04	1,03	-0,12
Tubulin alpha-1A chain	P68369	Tuba1a	1,41	-0,08	1,45	-0,05
Voltage-dependent anion-selective channel protein 1	Q60932	Vdac1	1,40	-0,02	1,05	-0,09
Latexin	P70202	Lxn	1,38	-0,37	1,63	-0,53

Protein name	Protein ID	Gene name	E1		E2	
			log_2 FC (TGFβ-1 /untr.)	log_2 FC (TGFβ-1 /TGFβ-1+E64d)	log_2 FC (TGFβ-1 /untr.)	log_2 FC (TGFβ-1 /TGFβ-1+E64d)
N(4)-(beta-N-acetylglucosaminyl)-L-asparaginase	Q64191	Aga	1,38	0,37	1,16	-0,07
Coactosin-like protein	Q9CQI6	Cotl1	1,33	0,02	1,77	-0,48
Cytochrome c oxidase subunit 6B1	P56391	Cox6b1	1,30	-0,05	0,88	-0,24
Cadherin-2	P15116	Cdh2	1,28	0,03	1,02	0,02
Cytochrome c oxidase subunit 2	P00405	Mtco2	1,27	0,01	0,96	-0,21
Calponin-3	Q9DAW9	Cnn3	1,27	-0,23	1,41	-0,49
Histone H4	P62806	Hist1h4a	1,26	-0,19	1,76	0,37
Tropomyosin alpha-1 chain	E9Q450	Tpm1	1,25	-0,25	0,92	-0,31
Cytochrome c oxidase subunit 4 isoform 1, mitochondrial	P19783	Cox4i1	1,24	-0,07	0,89	-0,17
Calponin-2	Q08093	Cnn2	1,23	-0,26	1,50	-0,49
Histone H3.1	E9PV77	H3f3a	1,22	-0,07	1,46	0,46
Cytochrome c oxidase subunit 5A, mitochondrial	P12787	Cox5a	1,22	0,02	1,01	-0,22
Prolow-density lipoprotein receptor-related protein 1	Q91ZX7	Lrp1	1,20	-0,17	1,48	0,10
Ferritin light chain 1	P29391	Ftl1;Ftl2	1,20	2,87	2,28	0,96
Unconventional myosin-Ib	Q7TQD7	Myo1b	1,19	-0,26	1,48	-0,07
N-acetylglucosamine-6-sulfatase	Q8BFR4	Gns	1,18	1,45	1,18	0,85
Fermitin family homolog 1	P59113	Fermt1	1,16	-0,16	1,22	-0,29
Mitochondrial carrier homolog 1	Q791T5	Mtch1	1,15	0,21	1,05	-0,21
Myosin-9	Q8VDD5	Myh9	1,14	-0,64	0,90	0,46
Thrombospondin-1	Q80YQ1	Thbs1	1,13	-0,38	0,91	-0,24
Cathepsin D	P18242	Ctsd	1,12	0,90	1,27	0,90
Phospholipase D3	O35405	Pld3	1,12	1,40	0,91	1,59
Sodium- and chloride-dependent taurine transporter	O35316	Slc6a6	1,11	0,36	1,15	0,08
Actin, alpha cardiac muscle 1 muscle	P68033	Actc1	1,10	-0,19	1,04	-0,40
Palladin	Q9ET54	Palld	1,09	-0,17	1,09	0,09
Protein Cald1	E9QA15	Cald1	1,08	-0,26	1,16	-0,27
Tropomyosin alpha-3 chain	E9Q5J9	Tpm3	1,07	-0,03	1,17	-0,29
Histone H2A.x;Histone H2A type 2-B	P27661	H2afx	1,04	-0,57	0,90	-0,45
Putative sodium-coupled neutral amino acid transporter 10	Q5I012	Slc38a10	1,03	-0,34	1,35	-0,34
Serpin H1	P19324	Serpinh1	1,03	-0,38	0,63	-0,07
Ras-related protein R-Ras	P10833	Rras	1,03	-0,27	0,72	-0,29
Xaa-Pro dipeptidase	Q11136	Pepd	1,02	0,24	1,04	0,05
Retinol-binding protein 1	Q00915	Rbp1	1,02	-0,21	1,64	-1,10
TSC22 domain family protein 1	E9QLZ1	Tsc22d1	1,02	-0,02	1,39	-0,36
Glutamate--cysteine ligase catalytic subunit	P97494	Gclc	1,01	-0,18	1,21	-0,37

Protein name	Protein ID	Gene name	E1		E2	
			log_2 FC (TGFβ-1 /untr.)	log_2 FC (TGFβ-1 /TGFβ-1+E64d)	log_2 FC (TGFβ-1 /untr.)	log_2 FC (TGFβ-1 /TGFβ-1+E64d)
Tropomyosin alpha-3 chain	E9Q7Q3	Tpm3	1,01	-0,03	1,11	-0,24
PDZ and LIM domain protein 7	Q3TJD7	Pdlim7	1,00	-0,25	0,73	-0,29
OX-2 membrane glycoprotein	O54901	Cd200	0,99	0,04	0,78	-0,20
Histone H2A type 1	P22752	Hist1h2ab	0,99	-0,38	1,07	-0,15
Microtubule-associated protein 4	P27546	Map4	0,97	-0,10	0,84	-0,05
Niemann-Pick C1 protein	O35604	Npc1	0,96	1,04	0,74	0,78
L-lactate dehydrogenase B chain	P16125	Ldhb	0,93	-0,09	0,83	-0,12
Transmembrane protein 43	Q9DBS1	Tmem43	0,93	-0,20	0,73	-0,06
Glutathione S-transferase omega-1	O09131	Gsto1	0,93	-1,26	0,92	-1,39
Histone H2B	Q921L4	Hist1h	0,92	-0,16	1,06	0,11
Putative phospholipase B-like 2	Q3TCN2	Plbd2	0,91	1,22	1,06	0,93
Malectin	D3Z1M3	Mlec	0,91	-0,24	0,66	-0,12
Sideroflexin-3	Q91V61	Sfxn3	0,90	-0,10	0,98	-0,32
Protein FAM3C	Q91VU0	Fam3c	0,89	0,17	1,20	0,08
Bcl-2 homologous antagonist/killer	O08734	Bak1	0,86	0,11	0,69	-0,15
Histone H2A.Z	P0C0S6	H2afz	0,85	-0,07	0,87	0,11
Leucyl-cystinyl aminopeptidase	Q8C129	Lnpep	0,84	1,40	0,77	1,16
Glutathione peroxidase 1	P11352	Gpx1	0,84	-0,43	1,60	-1,18
Tropomyosin alpha-4 chain	Q6IRU2	Tpm4	0,84	-0,08	0,86	-0,16
Synaptobrevin homolog YKT6	Q9CQW1	Ykt6	0,84	0,15	0,82	0,24
LIM domain and actin-binding protein 1	Q9ERG0	Lima1	0,83	-0,20	1,34	-0,14
Unconventional myosin-Ic	Q9WTI7	Myo1c	0,83	-0,07	1,61	-0,05
Band 4.1-like protein 2	O70318	Epb41l2	0,82	-0,03	0,62	-0,40
ER lumen protein retaining receptor 2	Q9CQM2	Kdelr2	0,82	0,17	0,70	0,02
Cysteine-rich protein 2	Q9DCT8	Crip2	0,79	-0,50	0,72	-0,63
Glutamate dehydrogenase 1, mitochondrial	P26443	Glud1	0,79	-0,09	0,74	-0,19
Zyxin	Q62523	Zyx	0,78	-0,08	0,82	-0,29
Very long-chain specific acyl-CoA dehydrogenase, mitochondrial	P50544	Acadvl	0,77	-0,19	0,60	-0,26
Beta-hexosaminidase subunit beta	P20060	Hexb	0,76	1,02	1,01	-0,02
Surfeit locus protein 4	Q64310	Surf4	0,76	-0,06	0,70	-0,20
NADH dehydrogenase [ubiquinone] iron-sulfur protein 7, mitochondrial	Q9DC70	Ndufs7	0,75	-0,09	0,68	-0,37
Sulfated glycoprotein 1	Q61207	Psap	0,75	0,48	1,01	0,41
Prenylcysteine oxidase	Q9CQF9	Pcyox1	0,74	0,35	1,01	0,26
Vesicle-trafficking protein SEC22b	O08547	Sec22b	0,74	-0,06	0,75	-0,07
Inositol 1,4,5-trisphosphate receptor type 3	P70227	Itpr3	0,74	0,36	0,79	0,18
Integrin beta-1	P09055	Itgb1	0,74	0,23	0,69	0,23
Solute carrier family 25	Q8JZU2	Slc25a1	0,73	0,13	0,75	-0,06

Protein name	Protein ID	Gene name	E1		E2	
			log_2 FC (TGFβ-1 /untr.)	log_2 FC (TGFβ-1 /TGFβ-1+E64d)	log_2 FC (TGFβ-1 /untr.)	log_2 FC (TGFβ-1 /TGFβ-1+E64d)
Alpha-actinin-1	Q7TPR4	Actn1	0,72	-0,09	0,90	-0,19
Galectin-1	P16045	Lgals1	0,72	0,03	1,23	-0,37
Cytosolic phospholipase A2	P47713	Pla2g4a	0,71	-0,41	1,50	-0,97
Calpain-2 catalytic subunit	O08529	Capn2	0,69	0,01	0,67	-0,04
14-3-3 protein gamma	P61982	Ywhag	0,69	0,00	0,60	0,07
Ribosome-binding protein 1	Q99PL5	Rrbp1	0,69	-0,13	0,88	-0,08
Delta-1-pyrroline-5-carboxylate synthase	Q9Z110	Aldh18a1	0,67	-0,17	0,59	-0,32
Integrin beta-6	Q9Z0T9	Itgb6	0,67	0,45	0,80	0,37
Supervillin	E9PVA9	Svil	0,65	-0,34	0,60	-0,42
Thioredoxin domain-containing protein 5	Q91W90	Txndc5	0,64	-0,13	0,62	-0,04
Calcium-binding mitochondrial carrier protein SCaMC-1	Q8BMD8	Slc25a24	0,63	0,00	0,85	-0,31
Rab GDP dissociation inhibitor alpha	P50396	Gdi1	0,63	-0,03	1,01	-0,12
Ladinin-1	P57016	Lad1	0,59	0,10	0,63	0,00
ELAV-like protein 1	P70372	Elavl1	0,58	-0,11	0,66	0,23
Protein Utrn	E9Q6R7	Utrn	0,58	-0,17	0,68	-0,15

Table S2: List of all proteins with higher abundance upon TGFβ-1 treatment of iPL32 identified in the quantitative proteome comparison: All proteins with log_2 fold change (Fc) ratios TGFβ-1/untreated ≥ 0.58 (highlighted in grey) in both experiment1 "E1" and experiment2 "E2" are shown with proteins most altered on top. The respective log_2 FC ratios TGFβ-1/TGFβ-1+E64d are also shown. Lysosomal proteins as determined by KEGG are highlighted in blue.
