# Supplementary material for: Lysosomal protein turnover contributes to the acquisition of TGFβ-1 induced invasive properties of mammary cancer cells
Source: Mol Cancer. 2015 Feb 15;14:39. doi: 10.1186/s12943-015-0313-5 (PMC4339013; doi:10.1186/s12943-015-0313-5)
Supplement: Additional file 5: Table S3. — List of proteins with altered abundance upon cysteine cathepsin inhibition in TGFβ-1 treated iPL32 cells identified in the quantitative proteome comparison: All proteins at least 50% higher or lower upon E64d treatment (log2 Fc TGFβ-1/untr. ≤ −0.58 or ≥ 0.58, highlighted in grey) in both experiment 1 “E1” and experiment 2 “E2” are shown. The respective log2 FC ratios TGFβ-1/untr. are also shown. [file 12943_2015_313_MOESM5_ESM.pdf]

- Supplementary Information Table S3 -

Proteins with altered abundance upon E64d treatment

Protein name	Protein ID	Gene name	E1		E2	
			log_2 FC (TGFβ-1 /untr.)	log_2 FC (TGFβ-1 /TGFβ-1+E64d)	log_2 FC (TGFβ-1 /untr.)	log_2 FC (TGFβ-1 /TGFβ-1+E64d)
less abundant upon E64d treatment:						
Glutathione S-transferase omega-1	O09131	Gsto1	0.93	-1.26	0.92	-1.39
Heterogeneous nuclear ribonucleoprotein M	Q9D0E1	Hnrnpm	0.14	-0.83	-0.06	-1.24
YLP motif-containing protein 1	D3YWX2	Ylpm1	0.14	-0.86	0.05	-0.98
ATP-dependent RNA helicase DDX3X	Q62167	Ddx3x	-0.26	-0.81	-0.16	-0.96
Probable ATP-dependent RNA helicase DDX5	Q8BTS0	Ddx5	-0.11	-0.73	-0.03	-0.66
Collagen alpha-1(XII) chain	Q60847	Col12a1	3.04	-1.16	2.32	-0.65
more abundant upon E64d treatment:						
Cathepsin L1	P06797	Ctsl1	0.38	3.75	0.57	2.33
Ferritin light chain 1	P29391	Ftl1	1.20	2.87	2.28	0.96
Junctional adhesion molecule A	O88792	F11r	0.22	2.09	0.31	1.63
Amyloid beta A4 protein	P12023	App	0.20	2.05	0.70	1.09
Acid ceramidase	Q9WV54	Asah1	0.51	1.70	0.46	1.55
CD63 antigen	P41731	Cd63	0.47	1.65	0.66	1.70
Dual specificity protein phosphatase 3	B1AQF4	Dusp3	0.10	1.59	-0.01	0.64
Lysosomal alpha-glucosidase	P70699	Gaa	0.48	1.51	0.46	1.81
Lysosome-associated membrane glycoprotein 2	P17047	Lamp2	0.41	1.49	0.51	1.23
Lysosome-associated membrane glycoprotein 1	P11438	Lamp1	0.42	1.49	0.28	1.36
N-acetylglucosamine-6-sulfatase	Q8BFR4	Gns	1.18	1.45	1.18	0.85
Phospholipase D3	O35405	Pld3	1.12	1.40	0.91	1.59
Leucyl-cystinyl aminopeptidase	Q8C129	Lnpep	0.84	1.40	0.77	1.16
Transferrin receptor protein 1	Q62351	Tfrc	-0.84	1.36	-0.85	1.84
Interferon-induced transmembrane protein 2	Q99J93	Ifitm2	0.52	1.28	1.10	0.66
Epididymal secretory protein E1	Q9Z0J0	Npc2	0.38	1.23	0.67	0.65
Putative phospholipase B-like 2	Q3TCN2	Plbd2	0.91	1.22	1.06	0.93
Lysosome membrane protein 2	O35114	Scarb2	0.38	1.19	0.39	0.79
Cathepsin B	P10605	Ctsb	0.46	1.10	0.45	0.89
Bleomycin hydrolase	Q8R016	Blmh	-0.33	1.07	-0.45	1.28
Niemann-Pick C1 protein	O35604	Npc1	0.96	1.04	0.74	0.78
Thioredoxin-related transmembrane protein 1	Q8VBT0	Tmx1	0.12	1.03	-0.09	1.16
Cathepsin D	P18242	Ctsd	1.12	0.90	1.27	0.90
Galectin-3	P16110	Lgals3	-1.38	0.84	-2.29	1.68

Protein name	Protein ID	Gene name	E1 log$_2$ FC (TGFβ-1 /untr.)	E1 log$_2$ FC (TGFβ-1 /TGFβ-1+E64d)	E2 log$_2$ FC (TGFβ-1 /untr.)	E2 log$_2$ FC (TGFβ-1 /TGFβ-1+E64d)
more abundant upon E64d treatment:						
ADP-ribosylation factor-like protein 8B	Q9CQW2	Arl8b	0.33	0.82	0.46	0.72
Glucosamine-6-phosphate isomerase 1	O88958	Gnpda1	0.46	0.80	0.66	0.60
Ras-related GTP-binding protein D	B1AWT3	Rragd	0.26	0.78	0.21	0.63
Proteasome subunit beta type-7	P70195	Psmb7	-0.30	0.77	-0.26	0.64
Synaptic vesicle membrane protein VAT-1 homolog	Q62465	Vat1	0.44	0.76	0.23	0.83
V-type proton ATPase subunit E 1	P50518	Atp6v1e1	-0.04	0.73	-0.24	0.72
Vesicle-associated membrane protein 8	O70404	Vamp8	0.32	0.68	0.20	0.99
V-type proton ATPase catalytic subunit A	P50516	Atp6v1a	-0.06	0.68	-0.29	0.72
Proteasome subunit beta type-2	Q9R1P3	Psmb2	-0.18	0.67	-0.20	0.74
Proteasome subunit beta type-4	P99026	Psmb4	-0.08	0.67	-0.10	0.75
Ras-related protein Rab-7a	P51150	Rab7a	0.32	0.67	0.26	0.72
5-nucleotidase	Q61503	Nt5e	1.95	0.67	1.73	0.60
Inositol monophosphatase 1	Q924B0	Impa1	-0.12	0.66	-0.27	0.80
Proteasome subunit alpha type-3	O70435	Psma3	-0.11	0.64	-0.08	0.73
Toll-interacting protein	Q9QZ06	Tollip	-0.07	0.63	-0.10	0.67
V-type proton ATPase subunit G 1	Q9CR51	Atp6v1g1	-0.02	0.62	-0.16	0.69
Proteasome subunit beta type-5	O55234	Psmb5	-0.11	0.62	-0.05	0.64
Proteasome subunit alpha type-1	Q9R1P4	Psma1	-0.21	0.62	-0.21	0.85
Proteasome subunit alpha type-6	Q9QUM9	Psma6	-0.13	0.62	-0.06	0.69
Proteasome subunit beta type-3	Q9R1P1	Psmb3	-0.12	0.62	-0.02	0.60
Lactadherin	P21956	Mfge8	-0.82	0.61	-0.11	0.65
Ras-related protein Rab-9A	A2AFP5	Rab9	-0.09	0.60	-0.43	0.74
V-type proton ATPase subunit B	P62814	Atp6v1b2	-0.18	0.60	-0.34	0.60
Nicastrin	F6UF97	Ncstn	0.30	0.59	0.30	0.66
Proteasome subunit beta type-6	Q60692	Psmb6	-0.18	0.59	-0.16	0.69

Table S3: **List of proteins with altered abundance upon cysteine cathepsin inhibition in TGFβ-1 treated iPL32 cells identified in the quantitative proteome comparison:** All proteins at least 50% higher or lower upon E64d treatment (log$_2$ Fc TGFβ-1/untr. ≤ -0.58 or ≥ 0.58, highlighted in grey) in both experiment 1 "E1" and experiment 2 "E2" are shown. The respective log$_2$ FC ratios TGFβ-1/untr. are also shown.
